# Inhibition of Microbial Growth and Biofilm Formation in Pure and Mixed Bacterial Samples

**DOI:** 10.3390/microorganisms12071500

**Published:** 2024-07-22

**Authors:** John D. Cate, Young Z. Sullivan, Maria D. King

**Affiliations:** Biological and Agricultural Engineering, Texas A&M University, College Station, TX 77843, USA; johncate@tamu.edu (J.D.C.); young.zheng@yahoo.com (Y.Z.S.)

**Keywords:** back-produced fracturing water, bacteria, sessile, planktonic, biofilm, microbiome, natural antimicrobials

## Abstract

Hydraulic fracturing, or fracking, requires large amounts of water to extract fossil fuel from rock formations. As a result of hydraulic fracturing, the briny wastewater, often termed back-produced fracturing or fracking water (FW), is pumped into holding ponds. One of the biggest challenges with produced water management is controlling microbial activity that could reduce the pond water’s reusable layer and pose a significant environmental hazard. This study focuses on the characterization of back-produced water that has been hydraulically fractured using chemical and biological analysis and the development of a high-throughput screening method to evaluate and predict the antimicrobial effect of four naturally and commercially available acidic inhibitors (edetic acid, boric acid, tannic acid, and lactic acid) on the growth of the FW microbiome. Liquid cultures and biofilms of two laboratory model strains, the vegetative *Escherichia coli* MG1655, and the spore-forming *Bacillus atrophaeus* (also known as *Bacillus globigii*, BG) bacteria, were used as reference microorganisms. Planktonic bacteria in FW were more sensitive to antimicrobials than sessile bacteria in biofilms. Spore-forming BG bacteria exhibited more sensitivity to acidic inhibitors than the vegetative *E. coli* cells. Organic acids were the most effective bacterial growth inhibitors in liquid culture and biofilm.

## 1. Introduction

Hydraulic fracturing, or fracking, is a widely employed multistep extraction technology to significantly increase oil and natural gas production by drilling and fracturing fossil-fuel-bearing rock formations in deep (over 2 km) shale reservoirs using large amounts of water. While the fracturing process has expanded oil and gas development by exploiting earlier inaccessible reserves, it has also added risks to water resources. Although numerous studies focus on the fracturing process and its effect on the environment, little is known currently about the effect of biocides on the formation of biofilm in the fracturing water and its microbiome composition. This study aims at addressing this gap by testing the effect of four different antimicrobials on the mixed bacterial liquid culture and biofilm of FW in comparison to a vegetative and spore-forming bacterial culture and biofilm.

Control of biofouling and biofilms in the oil industry is of great importance [1]. Within oil reservoirs where bacteria and viruses are abundant, biofilm formation enhances nutrient uptake, syntrophic interactions, and protection against environmental stress [2]. Liu et al. [3] found that microbiome composition in the oil field was strongly affected by environmental factors, such as temperature, oxygen content, salinity, and pH, which could be altered due to oil production. Microbial enhanced oil recovery (MEOR) is a promising substitute for other enhanced oil recovery methods in terms of sustainable development [4]. The effects of increasing discharge of treated shale gas wastewater on the microbial community in the receiving water had no significant effects on alpha diversity in the two wet seasons but had significant effects in the dry season after 15 months of discharge [5]. The number of strains with antibiotic resistant genes increased in relative abundance at the downstream site near the outfall.

Delineating the microbiome composition using 16S rRNA sequencing and Minimum Inhibitory Concentration (MIC) of the standard biocides in the produced water samples [6] show important taxonomy differences but similar functional characterization. The study indicates the high diversity of the microbiomes with varying resistance levels against the biocides, suggesting the need for customized biocidal treatments in oil fields. However, as many biocides are short-lived or degradable through abiotic and biotic processes, but some may transform into more toxic or persistent compounds, understanding the fate of biocides under downhole conditions (high pressure, temperature, and salt and organic matter concentrations) is limited [7]. While some biocides used to mitigate microbially induced corrosion and gas souring have been identified as toxic to humans and the environment, the selective antimicrobial pressure they cause has not been considered seriously [8]. Fracturing practices could potentially create antimicrobial resistance hotspots that are largely unknown in the literature, practice, and regulatory agencies. In the extreme environment of oil reservoirs, the distribution and abundance of antibiotic resistance genes (ARGs) remains poorly understood. The study of [9] found that ARGs were present in all parts of a water-flooded oilfield in China, with sulfonamide resistance as the most abundant. In the oil and gas industry, MIC causes up to 20–40% of serious corrosion cases and up to 70–95% of pipeline leaks [10]. This damage drives the market for corrosion inhibitors [11,12]. Environmental regulatory changes are phasing out inorganic inhibitors, driving innovation in organic based alternatives. However, biobanks and collections of biofilm samples relevant to industrial applications are lacking to address challenges [13].

Biofilm formation in shale gas fracturing flowback and the production of water reservoirs due to microbial contamination are growing environmental concerns. With increasing oil production, the original 500 bbl (oil barrels, equal to 21,000 gal or 79,500 L) fracturing water tanks have been gradually replaced by lined or unlined earthen pits, which are open to the ambient, including bioaerosols, dust, rain, and surface water. These source ponds are highly contaminated with bacteria [14] that can be unique to fracturing operations, including the recently discovered *Candidatus* Frackibacter [15,16]. During the fracturing process, different EPA-approved biocides (glutaraldehyde, quaternary ammonium chloride, sodium hypochlorite, THPS (tetrakis hydroxymethyl phosphonium sulfate), DBNPA (2,2-dibromo, 3-nitriloproprionamide)) are used to sanitize treatment water. However, mixing fracturing waters from different sources and reusing flowback waters with high concentrations of salts and metals [17] can cause elevated bacterial contamination as bacteria survive and establish communities resistant to biocides. Some microbes make glycine betaine that protects cells against osmotic stress from the shale’s high salt content. Other microbes can produce sulfides, leading to equipment corrosion [16]. Many studies focus on mitigating biofilm formation in the oil industry to prevent microbiologically influenced corrosion [18].

A thorough understanding of system operations and the delineation of microbial interactomes is required to properly design a bacterial control program and select biocide treatments for the effective control of the heterogeneous microbiome that will be present, including corrosive sulfate-reducing and acid-producing bacteria, algae, and fungi [14,19,20,21,22]. Gene cone libraries based on 16S rRNA for flowback water microbiome revealed diverse, depth-dependent communities belonging to several taxa, including *Proteobacteria* [23]. Archaea, specifically the class *Methanomicrobia*, were identified only in the untreated and biocide-amended impoundments. Metagenomic sequencing showed increased anaerobic classes in produced water compared to aerobic bacteria in the source water [24]. The results also suggest that microbial communities in fracturing water have increased genetic ability to handle stress. A comprehensive study based on 16S rRNA sequencing identified microbes that strive under high salinity and degrade hydrocarbons in fracturing water with complex inorganic and organic content [25].

Vikram et al. [26] demonstrated enhanced tolerance against glutaraldehyde, a biocide included in the fracturing water additive Alpha 1427, and increased susceptibility to hypochlorite in bacteria due to the salinity of the produced water. Another study of Vikram et al. [27] found that the composition of the active microbial community in produced water based on metatranscriptome analysis differed from that identified by 16S rRNA sequencing, which should be considered when selecting biocide application strategies. The attachment of free-floating bacteria to surfaces leads to multilayer biofilm formation with increased tolerance to biocides [14]. Hunt et al. [28] suggest that nutrient starvation may trigger biofilm detachment; another study found that inoculation density and nutrient availability determine the shape of biofilms [29].

In the review of Kahrilas et al. [30], the authors found that many biocides are short-lived or degradable, potentially transforming into more toxic or persistent compounds with limited knowledge available about their fate in downhole conditions. Li et al. [31] demonstrated that some D-amino acids could be used as biocide enhancers to reduce microbiologically influenced corrosion (MIC) in the oil and gas industry.

Several compounds (boric acid, EDTA, lactic acid and tannic acid) that are known for their quorum quenching, biocide, and antibiofilm activity are available in large quantities, naturally or commercially, and have been used in production processes from the food industry to water treatments [32]. Shefner and Burkhardt [33] delineated the toxic effect of boron from the pH effect, correlating it to the inhibition of xanthine oxidase [34,35,36]. Known for forming complexes with metals, the commercially available Ethylene Diamine Tetra Acetic Acid (EDTA, also known as edetic acid) can also play a significant role in antimicrobial treatments by scavenging ions that are essential to bacterial metabolism, impairing bacterial growth [37,38]. The antimicrobial effect of lactic acid bacteria in food preservation processes is mainly due to the acidic conditions they create, resulting in increased shelf-life and safety [39,40,41]. The efficacy of tannic acid has long been known among the common antimicrobial plant chemicals [42,43]. The inhibitory effect of tannic acid on the growth of intestinal bacteria may be due to the strong iron binding capacity of tannic acid [44], forming ferric tannate, which has been used to prevent rusting [45] or in leather treatment [46] due to its ability to inactivate microbial enzymes and proteins [47,48].

There is a dramatic difference in the effect of biocides, whether the treatment targets planktonic bacteria in liquid cultures or sessile microbes in expanding biofilms [49,50]. Although bacteria in biofilms are more resistant to antibiotics, boric acid is known to disrupt fungal and bacterial biofilms [51,52]. Similarly, the metal chelator EDTA has been shown to disrupt *P. aeruginosa* biofilm [53]. Payne et al. [54] presented that tannic acid inhibits *S. aureus* biofilm formation via a mechanism dependent upon a peptidoglycan hydrolase essential for cell wall growth and division.

The objective of this study is to characterize back-produced water that has been hydraulically fractured using chemical and biological analysis and develop a 96-well plate assay for high throughput, rapid assay to determine the antimicrobial effect of different concentrations of four naturally occurring and commercially available compounds that could potentially inhibit microbial growth and biofilm formation in fracturing water reservoirs. For comparison two laboratory strains, fresh vegetative bacterial cells of the Gram-negative *Escherichia coli* MG1655 and dry spores of the Gram-positive *Bacillus atropheaus* were used as model microorganisms.

## 2. Materials and Methods

### 2.1. Microbial Samples and Growth Medium

Back-produced fracturing water (FW) was collected from a cargo tank carrier upon arrival at the Eagle Ford Shale storage tank facility in Snook, TX, USA. Approximately 98% of the fracturing fluid is comprised of water and sand as proppant. In addition, acids, disinfectants, anti-corrosive agents, clay stabilizers, cross-linkers, friction reducers, non-emulsifiers, gelling, iron-controlling, and pH-adjusting agents have also been used (2%).

Two different bacterial strains, fresh mid-log phase *Escherichia coli* MG1655 cells and dry *Bacillus atrophaeus* (also known as *Bacillus globigii*, BG) spores, were used as reference cultures in the antimicrobial treatments.

The mid-log phase (OD_600_ = 0.5) fresh cultures of vegetative *Escherichia coli* K-12 MG1655 (*E. coli* Genetic Resources at Yale CGSC, The Coli Genetic Stock Center, New Haven, NE, USA) were grown in Luria Bertani (LB) medium [55] for about three hours at 37 °C with constant shaking at 0.102× *g*. The mid-log cells are uniform in age, size, and physiological characteristics, which allow them to respond more uniformly to the different treatments during testing [56]. The cells were harvested by pelletizing them at 2880× *g* for 7 min and resuspending them in LB. The final suspension had about 10^8^ cells/mL concentration.

A fresh batch of spore stock suspension (50 mg of dry BG powder in 10 mL of sterile Milli-Q water) was used for each set of sample tests. The dry BG spores were obtained from the Aerosol Sciences Laboratory of the US Army Chemical Biological Center (Aberdeen Proving Ground, MD). To remove traces of the original culture medium and cell debris, the spore suspension was vortexed for 5 min and harvested by pelleting in a centrifuge at 2880× *g* for 7 min, then resuspended in Milli-Q water. This procedure was repeated three times, finally resuspending the pellet in an LB medium at about 10^8^ spores/mL concentration. Of the fracturing water and the microbial suspensions (*E. coli* cells and BG spores), 100 µL aliquots were added in 1×, 10×, 100×, and 1000× dilutions in LB to the 360 µL wells of the microtiter plates for the inhibitor testing.

### 2.2. Total Solids (TS) and Total Suspended Solids (TSS)

Dry matter content or total solids (TS) were measured according to the Standard Method 2540C (adapted from ASTM D2974; 1995) [57]. Total suspended solids (TSS) were determined by the Environmental Protection Agency (EPA) Method 160.2 using pre-weighed 47 mm glass fiber A/E filters (Pall, Waltham, MA, USA).

### 2.3. Gas Chromatography-Mass Spectrometry (GC-MS) Analysis of Back-Produced Fracturing Water

The fracturing water sample was derivatized after the ethyl acetate extracted sample was evaporated to dryness and reacted with N, O-Bis(trimethylsilyl)trifluoroacetamide (BSTFA) at 60 °C for 1 h. The derivatized sample was directly analyzed by GC/MS (DSQ II GCMS; Thermo Scientific, Waltham, MA, USA).

### 2.4. Inductively Coupled Plasma Mass Spectrometry (ICP-MS) Elemental Analysis of Back-Produced Fracturing Water

Total elemental composition was analyzed by inductively coupled plasma mass spectroscopy (ICP-MS) on a Perkin Elmer Nexion 300D spectrometer (Perkin Elmer, Waltham, MA, USA) according to the EPA method 6010C.

### 2.5. Microbial Plating and Fatty Acid Methyl Ester (FAME) Analysis

Appropriately diluted aliquots of the fracturing water were plated on Tryptic Soy Agar (TSA) plates and incubated at 37 °C. The four most frequently occurring colonies based on morphological characteristics were isolated and analyzed by the fatty acid methyl ester analysis microbial identification system, FAME (MIDI Inc., Newark, DE, USA) [58].

### 2.6. DNA Isolation for Illumina Sequencing

To delineate the microbiome in the recycled fracturing water, 50 mL aliquots of the fracturing water samples were pelleted at 2880× *g* for 10 min, and the pellets were subjected to genomic DNA isolation according to the alkaline lysis method of Zhou et al. [59] using PolyAcryl Carrier (PAC; Molecular Research Center Inc., Cincinnati, OH, USA). The DNA samples served as templates in the polymerase chain reaction (PCR) to amplify a 123 bp bacterial fragment located on the 16S rRNA using the oligonucleotides 1369F (5′-CGG TGA ATA CGT TCY CGG) and 1492R (5′-GGT TAC CTT GTT ACG ACT). Each PCR reaction contained 1× ThermoPol reaction buffer (NEB, Ipswich, MA, USA), 0.025 units/µL ThermoPol Taq polymerase, 0.8 mM dNTP mixture, 1.0 µg/µL BSA, 200 pM of each primer and 0.15–0.5 ng genomic DNA as template. The 16S thermo-cycling conditions were 94 °C for 5 min, 40 cycles of 94 °C for 30 s, 60 °C for 30 s, 72 °C for 30 s, and finally 72 °C for 10 min. The amplicons were submitted for Illumina sequencing, and the results were evaluated by the QIIME program [60].

### 2.7. Inhibitory Effect of Antimicrobials on Microbial Growth and Biofilm Formation

Four antimicrobial compounds were selected to study their inhibitory effect on the fracturing water. Based on the presence of both Gram-positive and Gram-negative strains in the fracturing water, two model bacteria, the Gram-positive *Bacillus atrophaeus*, also known as *B. globigii* (BG) (0.06 mg/mL; 23,500,000 CFU/mL) and the Gram-negative *Escherichia coli* MG1655 (12,300,000 CFU/mL) were selected for comparison in the treatments. The effect of the four antimicrobials on bacterial growth and biofilm formation in fracturing water and the two model bacteria was tested in 96-well polystyrene plates with maximum well volumes of 360 μL. The aqueous solution of an inorganic compound, boric acid (Sigma, St. Louis, MO, USA), was used in increasing concentrations (0 g/L, 100 mg/L, 250 mg/L, 500 mg/L, 1 g/L, and 5 g/L) in the experiments to test for inhibitory activity. In addition, three organic compounds, Ethylene Diamine Tetra Acetic Acid, Sodium salt (EDTA), tannic acid (TA), and lactic acid (LA) (Sigma, St. Louis, MO, USA) were also tested in the same concentrations (0 g/L, 100 mg/L, 250 mg/L, 500 mg/L, 1 g/L, and 5 g/L). Of each inhibitor solution, an equal volume (100 µL) was added to the different dilutions (1, 10×, 100×, and 1000×) of the FW and microbial suspensions in LB medium (100 µL) in the wells. For BG and *E. coli*, 1× (undiluted) to 1000× dilutions correspond from 2 × 10^7^ to 2 × 10^4^ CFU/mL culturable counts, respectively. The BG and *E. coli* plates were covered and incubated under sterile conditions and constant agitation at 0.102 g’s for 24 h at 37 °C for liquid culture growth inhibition and for 6 days at 37 °C under static conditions for biofilm inhibition.

The optical density of the 24 h liquid cultures in each well was read at 485 nm to maximize absorbance (due to the yellow color of LB in the samples) using the Tecan Infinite F500 microplate reader with Magellan Standard data analysis software (Tecan US Inc., Morrisville, NC, USA).

The relative turbidity for the liquid culture samples was calculated based on the formula:

Relative Turbidity = (Test OD_485 nm_)/(Control OD_485 nm_), where the Control had 0 g/L antimicrobial concentration.

After the six-day incubation, the biofilm plates were washed with sterile phosphate-buffered saline (PBS, pH 7.4) to remove the planktonic cells, and the biofilm was stained with 0.4% crystal violet. Wells containing untreated microbial suspensions in LB were used as controls; LB medium and antimicrobial solutions were used as blank samples. All assays were repeated at least three times, and results were calculated as relative turbidity.

### 2.8. Crystal Violet Assay

The crystal violet assay, based on the ability of the dye to stain DNA, was used to obtain quantitative information about the relative density of cells adhering to the multi-well cluster plates [61,62]. Upon solubilization, the amount of dye taken up by the biofilm monolayer was quantitated in the plate reader. After carefully removing the culture medium from the wells, the plates were gently washed with 0.2 mL/well Phosphate Buffer Saline (PBS) buffer warmed at least to room temperature. After carefully removing PBS and adding 40 µg/well (10 µL of a 0.4% solution) crystal violet to stain the biofilm layer remaining in the wells, the covered plates were incubated for 10 min at room temperature. The plates were washed carefully in two fresh batches of tap water so as not to lift off cells. Excess liquid was drained by placing the plates upside down on paper towels. Finally, 1% SDS (Sigma, St. Louis, MO, USA) was added to solubilize the stain. The plates were agitated on an orbital shaker until the color was uniform, with no areas of dense coloration in the bottom of the wells. The optical density of the biofilm in each well was read at 590 nm using the Tecan Infinite F500 microplate reader with Magellan data analysis software (Tecan US Inc., Morrisville, NC, USA).

The relative turbidity for the biofilm samples was calculated based on the formula:

Relative Turbidity = (Test OD_590 nm_)/(Control OD_590 nm_), where the control had 0 g/L antimicrobial concentration.

The biofilms were allowed to form for six days as studies show that it can be expected that a biofilm is able to reach maturity within a week of inoculation [63]. The inhibitory concentration was determined to be the lowest concentration, which produced a visible disruption of biofilm formation and a significant reduction in the optical density compared with the reference wells at OD 590 nm [62,63,64].

### 2.9. Statistical Analysis

Statistical analysis was performed using MATLAB functions and analysis of variance (ANOVA).

ANOVA analysis was conducted for the data where the relative turbidity value was <1, consisting of a subset of 356 observations. The experimental data were handled as a factorial design where bacteria, culture, antimicrobial (AM), AM dilution (AMdil), and bacterial dilution (Bdil) were treated as factors with absorbance as the response. A linear model was fitted to determine if the factors significantly affected the response.

## 3. Results

### 3.1. Total Solids (TS) and Total Suspended Solids (TSS)

The total solids content of the fracturing water was 7153 mg/L; total suspended solids were 2524 mg/L.

### 3.2. GC-MS Analysis of Fracturing Water

Figure S1 (Supporting Information) shows the chemical composition of the fracturing water. The main hydrocarbon components are the aliphatic Tridecane, Tetradecane, Pentadecane, Hexadecane, 2,6,10-trimethyl Pentadecane, Heptadecane, and Octadecane. Two aromatics, m-cresol and 1,3-Benenedicarboxylic acid, bis(2-ethylhexyl) ester, were identified. Although the sample was derivatized with BSTFA to render the components more volatile, the identity of most of the peaks remained unknown. After derivatization, three acids could be identified: butylamine, boric acid, and 2-hydroxy propanoic acid.

### 3.3. ICP-MS Elemental Analysis

The elemental composition of the back-produced fracturing water shows high levels of sodium (1 × 10^6^ ppb), chlorine (8 × 10^5^ ppb), calcium (8 × 10^4^ ppb), carbon (8 × 10^4^ ppb), and potassium (1 × 10^4^ ppb) ions (See Figure S2, Supporting Information).

### 3.4. Microbial Plating and FAME Analysis

Microbial plating of fracturing water resulted in numerous colonies with diverse morphology; large colonies (3 × 10^4^ CFU/mL), medium colonies (5 × 10^4^ CFU/mL), and small colonies (2 × 10^4^ CFU/mL) were identified as actively growing cells on artificial media.

The five most frequently present culturable species on the growth plates were identified by FAME analysis as the Gram-negative, pleomorphic, metal-reducing bacterium *Shewanella putrefaciens*, the halophilic rod-shaped pathogen *Grimontia (Vibrio) hollisae*, the soil-dwelling pathogen *Methylobacterium (Methylorubrum) zatmanii*; the motile, short rod-shaped opportunistic pathogen *Serratia marcescens* and the rod-shaped *Pseudomonas fluorescens*-biotype G/*taetrolens*, dwelling in soil, plants, and water surfaces. All the five isolates belong to the phylum *Pseudomonadota* and class *Gammaproteobacteria*, except for *M. zatmanii* of the *Alphaproteobacteria* classification.

### 3.5. Microbiome of the Fracturing Water

**Figure 1:**
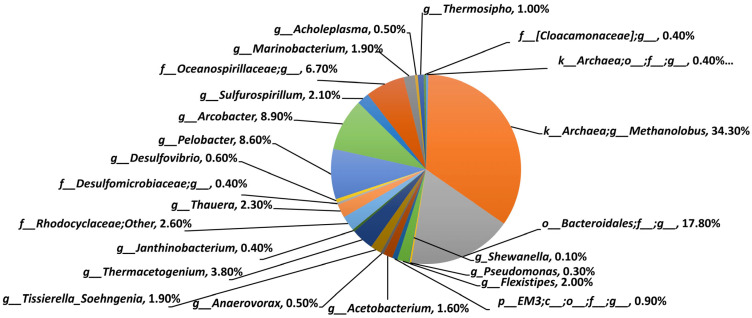
Illumina sequencing of the fracturing water microbiome composition showing the most frequently occurring 24 genera of the 68 identified strains. Abbreviations: k (kingdom), p (phylum), c (class), o (order), f (family), g (genus).

Illumina sequencing of the DNA sample extracted from the fracturing water shows the presence of a diverse microbiome (Figure 1).

The archea *Methanolobus* (highest percentage, 34.3%) is a coccoid methanogen growing only on methanol and methylamines. *Bacteroides luti* sp. *nov*. (10.5%) is an anaerobic, cellulolytic, and xylanolytic bacterium initially isolated from methanogenic sludge. An unusual *Arcobacter* species, designated strain CAB (8.9%), was isolated earlier from marine sediment and found to have the capacity to grow via perchlorate reduction, the only member of the *Epsilonproteobacteria* in pure culture to possess this rare metabolism. *Clostridium* (0.1%) from the phylum *Bacillota* and the *Gammaproteobacteria Oceanospirillaceae* (6.7%), *Marinobacterium* (1.9%), *Pseudomonas* (0.3%) and *Shewanella* (0.1%) are known hydrocarbon degraders and become enriched in the presence of crude oil [23]. Of the five culturable species identified by FAME, the genera *Shewanella* and *Pseudomonas* were also found in the FW microbiome. Similarly to the analysis by Mohan et al. [24], anaerobic/facultative anaerobic classes related to *Clostridia*, *Gamma*, and *Epsilonproteobacteria*, and *Bacteroidia* were also found in the produced fracturing water.

Table S1 (Supporting Information) shows the number of non-spore-forming (NS) and spore-forming (S) strains classified as Gram-negative (81% of total bacteria; 60% (NS) and 13.5% (S)) and Gram-positive (19% of total bacteria; 10% (NS) and 7.5% (S)) bacteria in the fracturing water used in this study.

### 3.6. Antimicrobial Treatments

#### 3.6.1. Boric Acid Treatments

**Figure 2:**
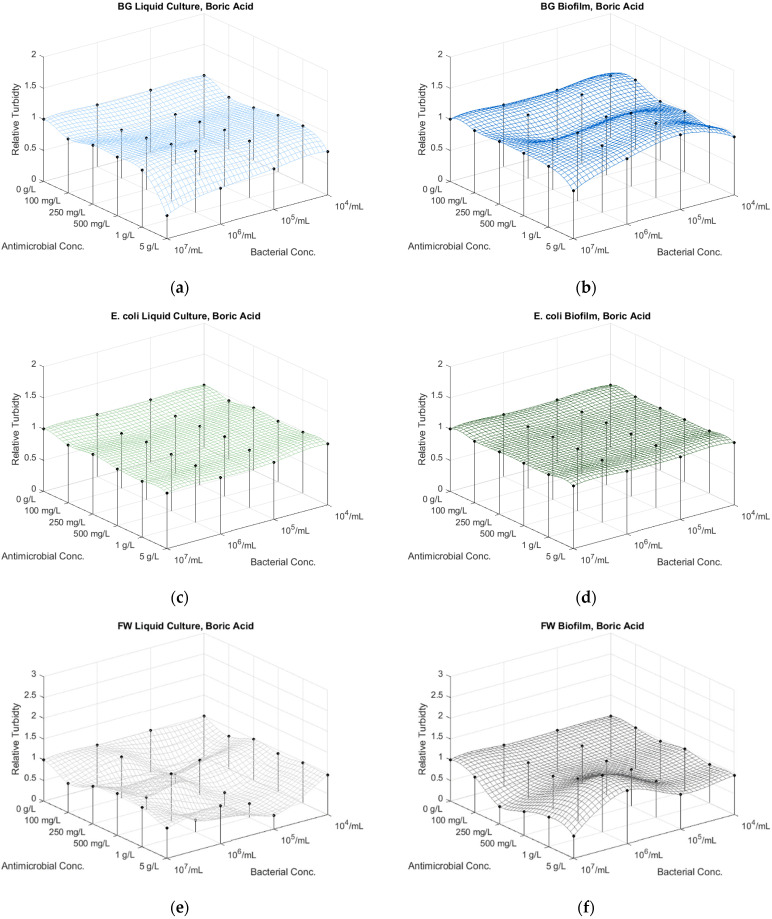
The effect of the concentration of antimicrobial agent boric acid and bacterial dilutions on the absorbance of the planktonic (liquid culture at 485 nm) and sessile (biofilm at 590 nm) of (**a**,**b**) *Bacillus atrophaeus* (BG), (**c**,**d**) *E. coli* and (**e**,**f**) FW bacteria.

The BG cultures responded uniformly to the boric acid treatment, showing some inhibition at all concentrations for the cultures in dilutions (Table S2a; Figure 2a and Figure S3a, Supporting Information). The 5 g/L boric acid concentration resulted in almost complete growth inhibition, even for the undiluted culture. The biofilm in all dilutions seemed to respond uniformly to all boric acid concentrations, with less sensitivity, however, with high standard deviation (Table S2b; Figure 2b and Figure S3b, Supporting Information).

Both the *E. coli* culture and biofilm responded uniformly without significant decrease in all dilutions to all inhibitor concentrations, exhibiting some sensitivity to pH changes (Table S2a,b; Figure 2c,d and Figure S4a,b, Supporting Information).

The bacterial concentrations in the FW samples decreased mostly at lower boric acid concentrations of 10^6^–10^5^ CFU/mL (Table S2a; Figure 2e and Figure S5a, Supporting Information). The standard error in the absorbance measurements may be related to the heterogeneous microbial populations that respond variably to decreasing pH values. The FW biofilm samples showed a similar tendency, albeit with less sensitivity to inhibitor concentration differences at lower bacterial concentrations 10^4^/mL (Table S2b; Figure 2f and Figure S5b, Supporting Information).

#### 3.6.2. EDTA Treatments

**Figure 3:**
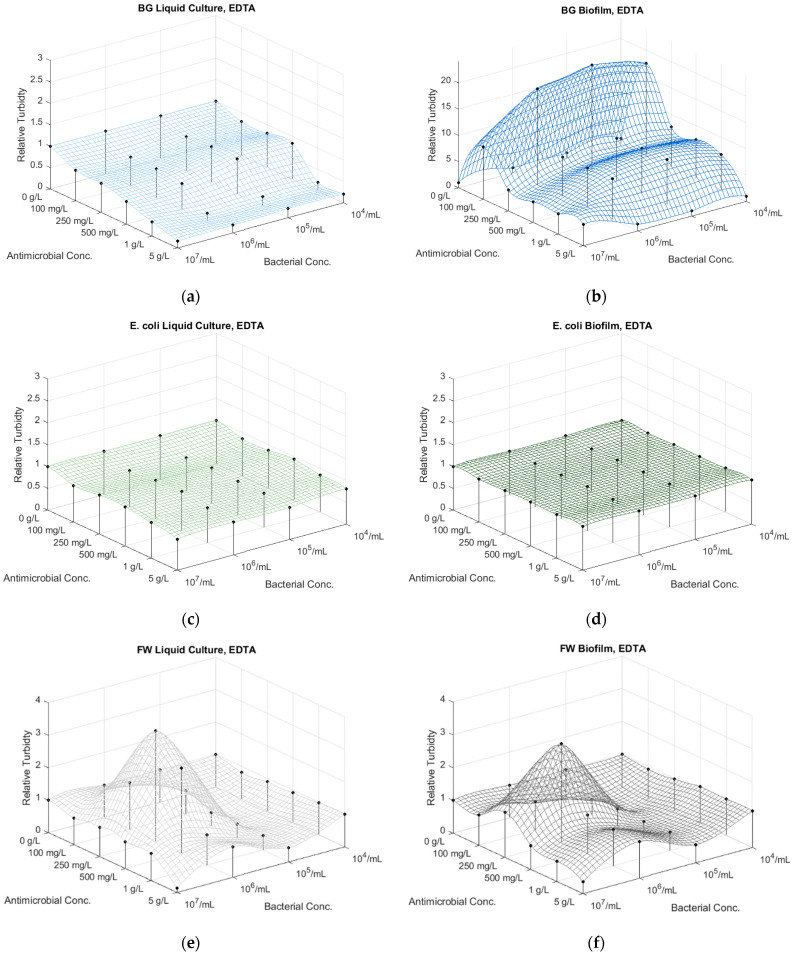
The effect of the concentration of antimicrobial agent edetic acid (EDTA) and bacterial dilutions on the absorbance of the planktonic (liquid culture at 485 nm) and sessile (biofilm at 590 nm) of (**a**,**b**) *Bacillus atrophaeus* (BG), (**c**,**d**) *E. coli* and (**e**,**f**) FW bacteria.

The BG culture showed a gradual decrease in culturability with increasing EDTA concentrations (Table S2a; Figure 3a and Figure S6a, Supporting Information). There was less sensitivity to increasing EDTA concentrations in the 100–500 mg/L range. However, a dramatic decrease is shown at 1 g/L, resulting in zero growth at bacterial dilutions above 10^6^ CFU/mL, and all dilutions at 5 g/L EDTA.

Interestingly, lower antimicrobial concentrations affected the BG biofilm formation positively until they reached the 5 g/L concentration, which was similarly critical for the diluted BG cultures below 10^7^ CFU/mL (Table S2b; Figure 3b and Figure S6a,b, Supporting Information). The 5 g/L EDTA concentration did not affect biofilm growth below 10^6^ CFU/mL concentrations.

Vegetative *E. coli* exhibited high tolerance to increasing EDTA concentrations, although there was an insignificant difference in growth between the untreated and treated cultures already at the lowest EDTA concentration (100 mg/L, Table S2a; Figure 3c and Figure S7a, Supporting Information). Similar lower relative turbidity values could be detected in the sessile bacteria with increasing EDTA concentrations. However, EDTA did not affect *E. coli* biofilm formation, showing insignificant difference between the untreated and treated cultures (Table S2b; Figure 3d and Figure S7b, Supporting Information).

The undiluted fracturing water liquid culture at 10^7^ CFU/mL did not respond negatively to EDTA concentrations below 5 g/L. However, it showed a positive effect at 10× dilution of FW at <1 g/L EDTA concentrations and retardation in growth at higher bacterial dilutions. EDTA inhibited growth at 100× culture dilutions (10^5^ CFU/mL), however, with no effect at lower dilutions (10^4^ CFU/mL) (Table S2a; Figure 3e and Figure S8a, Supporting Information). Similarly, the inhibitory effect of EDTA on FW biofilm was noticeable, resulting in growth suppression already at 100 mg/L EDTA concentrations for the 10^5^ CFU/mL dilutions, however, in higher relative turbidity at 250 mg/mL EDTA at the higher bacterial concentrations (Table S2b; Figure 3f and Figure S8b, Supporting Information). However, as the biofilm formation was slow, the staining and washing of the thin layer could have resulted in errors in the absorbance measurements.

#### 3.6.3. Lactic Acid Treatments

**Figure 4:**
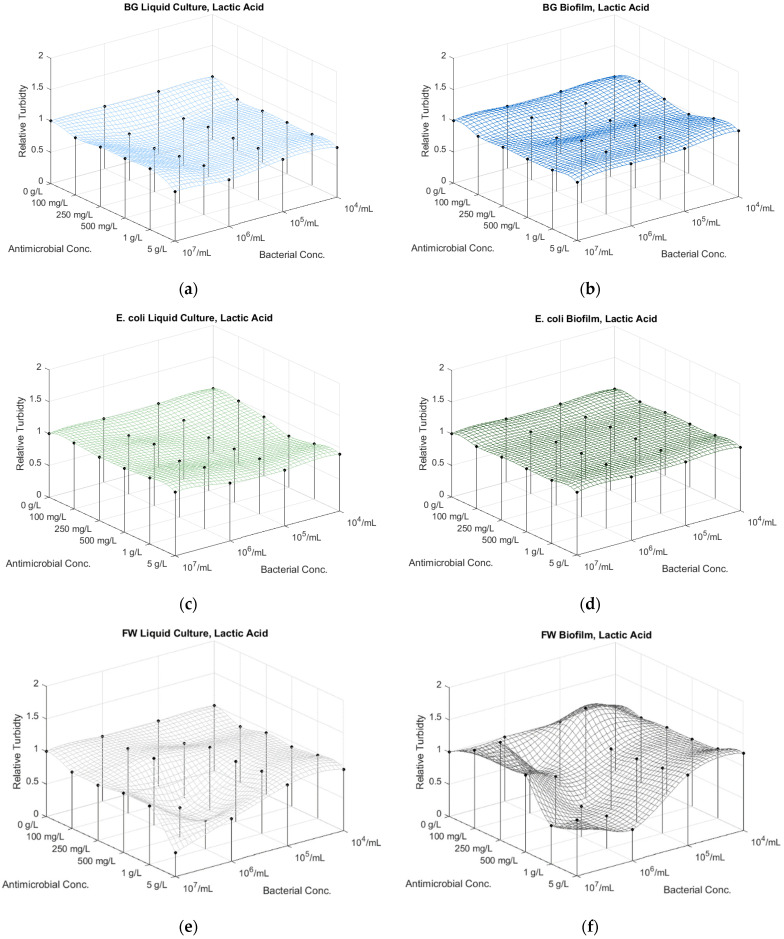
The effect of the concentration of antimicrobial agent lactic acid and bacterial dilutions on the absorbance of the planktonic (liquid culture at 485 nm) and sessile (biofilm at 590 nm) of (**a**,**b**) *Bacillus atrophaeus* (BG), (**c**,**d**) *E. coli* and (**e**,**f**) FW bacteria.

The lactic acid treatment of BG resulted in a decrease in culture growth at all inhibitor concentrations, more significantly from the 10x bacterial dilution (10^6^ CFU/mL) (Table S2a; Figure 4a and Figure S9a, Supporting Information). The biofilm responded more uniformly, exhibiting less sensitivity, and even growth at lower microbial concentrations (10^4^ CFU/mL) (Table S2b; Figure 4b and Figure S9b, Supporting Information).

For *E. coli*, the lactic acid treatment showed a uniform, increasing inhibition at higher bacterial dilutions and antimicrobial concentrations (Table S2a; Figure 4c and Figure S10a, Supporting Information). The biofilm exhibits almost no inhibition uniformly for all the dilutions (Table S2b; Figure 4d and Figure S10b, Supporting Information).

The lactic acid treatments resulted in an inhibitory effect for the FW concentrations 10^7^–10^6^ CFU/mL, however, exhibiting less decrease in culture growth at the higher FW dilutions < 10^5^ CFU/mL (Table S2a; Figure 4e and Figure S11a, Supporting Information).

The biofilm inhibitory effect shows a similar tendency at FW dilution: a decrease in turbidity at 10× FW dilutions, followed by an increase in turbidity at higher microbial dilutions (Table S2b; Figure 4f and Figure S11b, Supporting Information). Lactic acid concentrations lower than 500 mg/L seem to stimulate the growth of the undiluted FW biofilm.

#### 3.6.4. Tannic Acid Treatments

**Figure 5:**
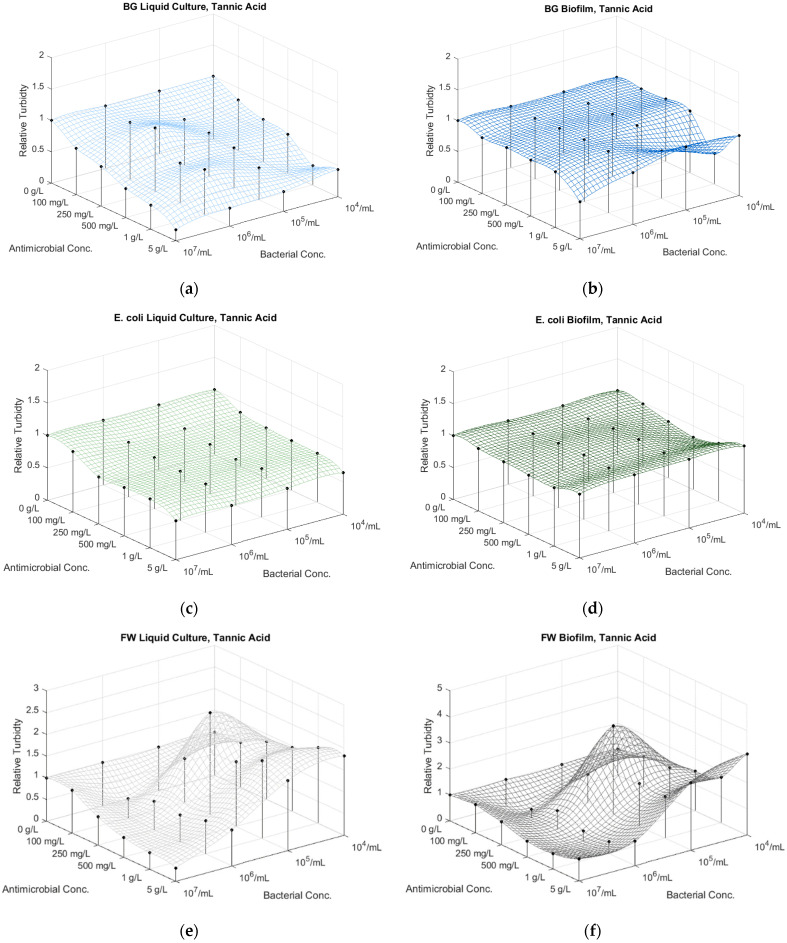
The effect of the concentration of antimicrobial agent tannic acid and bacterial dilutions on the absorbance of the planktonic (liquid culture at 485 nm) and sessile (biofilm at 590 nm) of (**a**,**b**) *Bacillus atrophaeus* (BG), (**c**,**d**) *E. coli* and (**e**,**f**) FW bacteria.

BG liquid culture responded with uniform sensitivity to the treatment, except for the 10× microbial dilution at lower tannic acid concentrations (Table S2a; Figure 5a and Figure S12a, Supporting Information). The biofilm exhibited less sensitivity at lower concentrations < 1–5 g/L, albeit with high standard deviation, maybe due to the dark pigmentation of the inhibitor offsetting the optical reader (Table S2b; Figure 5b and Figure S12b, Supporting Information).

The *E. coli* liquid culture samples showed similar tendency to the tannic acid treatments of the BG and FW cultures, with an increased background absorbance and inhibitory effect at higher tannic acid concentrations, especially at 5 g/L (Table S2a; Figure 5c and Figure S13a, Supporting Information). Barely any effect was exhibited for the biofilm values, except for the highest *E. coli* dilutions (10^4^ CFU/mL) (Table S2b; Figure 5d and Figure S13b, Supporting Information).

The dark pigmentation of the tannic acid presented a difficulty for absorbance measurements at higher inhibitor concentrations for the FW cultures, resulting in high background values at increased tannic acid concentrations (Table S2a; Figure 5e and Figure S14a, Supporting Information). The FW liquid culture and biofilm responded similarly to the tannic acid, with initial inhibition at higher microbial concentrations, followed by growth at higher microbial dilutions (100×–1000×) (Table S2b; Figure 5f and Figure S14b, Supporting Information).

### 3.7. ANOVA Analysis

ANOVA analysis of the results indicates that the bacterial dilution factor is not significant in the absorbance of the liquid cultures and biofilms. However, when the right ratio is applied between the concentration of the antimicrobial and the concentration of cells (dilution), it could result in a significant decrease in absorbance.

**Figure 6:**
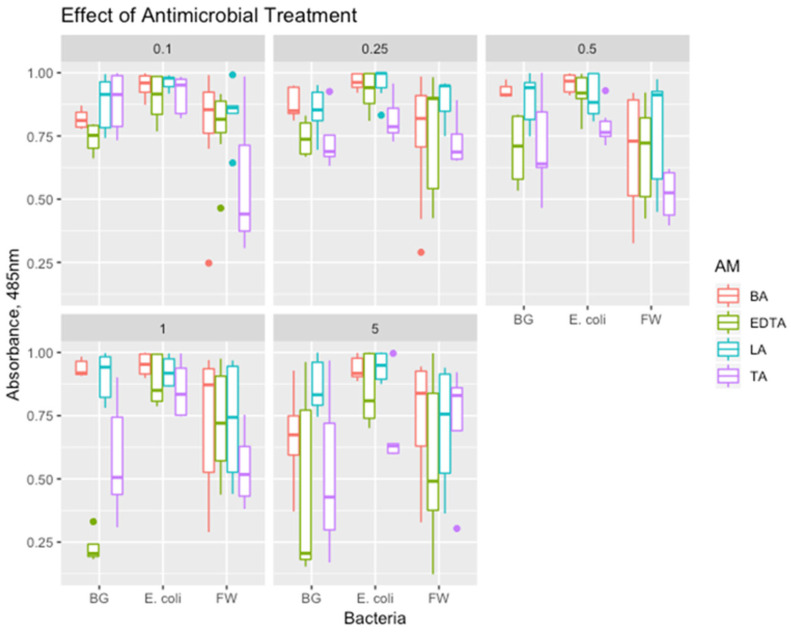
ANOVA analysis of the effect of the concentration of the antimicrobial (AM) agent on the absorbance of the BG, *E. coli* and FW liquid cultures (BA: boric acid; EDTA: edetic acid, LA: lactic acid; TA: tannic acid). The evaluation is based on experimental data in Table S2a (Supporting Information).

A boxplot of the data of absorbance versus bacteria (BG, *E. coli*, or FW) shows a trend of decreasing absorbance with increasing concentration (0.1 g/L–5 g/L) of the antimicrobial (AM) treatment (Figure 6). It is apparent that the antimicrobials were least effective on *E. coli*. Moreover, the FW bacteria and BG were the most susceptible to the antimicrobial agents, especially at higher concentrations.

## 4. Discussion

The four commercially available, naturally occurring bacterial growth inhibitors had different biocidal effects on the sessile and planktonic cells. Although bacterial spores are the sturdiest known life forms [65], BG spores during germination and growth responded more sensitively to acidic biocides than the vegetative bacterium *E. coli*. This sensitivity may be due to the presence of a significant permeability barrier to small molecules in the coat/outer membrane or other structure of intact spores [66,67]. Although spore germination has been studied for years, the mechanism underlying nutrient germination is not fully understood. Since intact spores respond well to low-molecular-weight germinants that reach the inner membrane (IM) from the medium, causing major change in IM permeability and structure, and leading to the release of monovalent cations, including H+, K+, and Na+ [68,69,70], this increased permeability may also enable the entry of acidic biocides.

Planktonic bacteria in FW exhibited more sensitivity to biocides than sessile bacteria in biofilms. Of the antimicrobials tested, the three organic compounds studied were more effective than inorganic boric acid, especially at higher planktonic content. Higher concentrations of EDTA and tannic acid showed a dramatic effect inhibiting planktonic BG bacteria, while tannic acid, at lower microbial concentrations, and lactic acid were the most effective inhibitors for *E. coli* liquid culture, with concentrations as low as 100 ppm detected to inhibit the growth of planktonic bacteria in a rich nutrient medium. The three organic compounds were more effective inhibiting biofilm growth in BG and FW bacteria at higher bacterial concentrations, while *E. coli* responded to tannic acid at lower microbial concentrations. Interestingly, liquid cultures of FW with EDTA and tannic acid exhibited growth at lower microbial concentrations, while boric acid and EDTA enhanced growth in the BG and FW biofilms. At lower microbial concentrations, the FW biofilm exhibited growth with lactic and tannic acid.

The biocidal effect of boric acid at the maximum concentration (5 g/L) is greater for the 10^7^ CFU/mL BG liquid culture compared to the dilution at 10^4^ CFU/mL. A similar trend has been observed for EDTA at the concentration of 500 mg/L and for tannic acid for the BG liquid culture. Boric acid at the lowest 100 mg/L concentration exhibits a greater biocidal effect for the mixed microbiome FW liquid culture compared to higher biocide concentrations (>250 mg/L). The liquid culture of FW at the highest bacterial concentration (10^7^ CFU/mL) reacts to the maximum concentrations (5 g/L) of EDTA, lactic acid and tannic acid; however, the effect is not noticeable for the diluted cultures (10^5^ CFU/mL and 10^4^ CFU/mL).

In these cases, the results for the antibacterial effect do not correlate linearly with the biocide and bacterial cell concentrations. This phenomenon could be explained at the molecular level. The capacity of a bacterial cell to cope with low pH stress is determined by its specific genes, encoding acid-induced proteins that can create altered molecular composition in the cell by structural and metabolic changes. While acidic pH can have a strong effect on the overall growth and behavior at the cellular level, within a large population of cells (in this study, for 10^7^ CFU/mL), acids can cause genetic and biochemical heterogeneity resulting in different behaviors. Within a mixed microbiome community such as FW, both the population structure and inter-species and intra-species interactions can be strongly influenced by changes in pH [71].

In summary, the microbiome of a back-produced fracturing water (FW) containing mostly aliphatic and significantly less aromatic or acidic compounds was delineated. Although the dominant strain (34%) *Methanolobus* is an Archaea, most bacteria in the FW microbiome are hydrocarbon degraders of the Phylum *Proteobacteria*. Of the total genera delineated in the FW, 81% are Gram-negative, and 19% are Gram-positive bacteria, including vegetative (60% and 10%) and spore-forming strains (13.5% and 7.5%), respectively. Although there is a difference between culturable bacteria and bacteria by sequencing as only about 2% of environmental bacteria are culturable in a laboratory setting [72], of the five culturable species identified by FAME, the genera *Shewanella* and *Pseudomonas* were also found in the FW microbiome. A 96-well plate assay for high throughput screening was developed for the rapid testing of the inhibition of planktonic and sessile bacterial growth in FW and two laboratory strains, the vegetative *E. coli* and spore-forming *B. atrophaeus* (BG). The antimicrobials were least effective on *E. coli*, while FW bacteria and BG were the most susceptible to the antimicrobial agents, especially at higher concentrations. The planktonic bacteria in FW were more sensitive to inhibitors than the sessile bacteria in biofilms while spore-forming BG bacteria exhibited more sensitivity to acidic inhibitors than the vegetative *E. coli* cells. EDTA seems to have enhanced growth in BG biofilm in sublethal (1 g/L and lower) concentrations and for both growth forms (liquid culture and biofilm) of FW microbiome at higher bacterial concentrations. EDTA at lower concentrations seems to enhance biofilm growth in BG by a specific mechanism that is not known. It is possible that the chelating agent EDTA combines with a cation present in a concentration that is inhibitory to the BG biofilm or can be replaced by another cation from the growth medium. This phenomenon may be compared to the behavior of *Bacillus anthracis*, the pathogenic strain for which BG is a surrogate, when utilizing e.g., thallium in the PLET medium that contains EDTA and thallous acetate, for the recovery of *B. anthracis* strains while inhibiting *B. cereus* [73]. Organic acids were found to be effective bacterial growth inhibitors in liquid culture and biofilm.

## 5. Conclusions

Numerous questions are yet to be addressed regarding the uncertainties of bacterial responses to natural antimicrobials and their efficient analysis. Future research will identify more potential biocides and the mechanism of their action. These natural antimicrobials could present an environmentally friendly solution for using biocides in fracturing and other industrial operations.

## Data Availability

Data are contained within the article and Supplementary Materials.

## Supplementary Materials

The following supporting information can be downloaded at: https://www.mdpi.com/article/10.3390/microorganisms12071500/s1, The classification of the genera delineated in the fracturing water microbiome is shown in Table S1. The results of the biocide treatments for the different bacterium (BG, *E. coli,* FW) liquid cultures are shown in Table S2a, for the bacterial biofilms in Table S2b. Detailed graphs showing the results for the GC-MS analysis (Figure S1) and the ICP-MS elemental analysis (Figure S2) of the fracturing water and for the boric acid, edetic acid, lactic acid and tannic acid treatments (Figures S3–S14) are available in the Supporting Information (SI).

## References

[1] Tiburcio S.R.G., Macrae A., Peixoto R.S., da Costa Rachid C.T.C., Mansoldo F.R.P., Alviano D.S., Alviano C.S., Ferreira D.F., de Queiroz Venâncio F., Ferreira D.F. (2021). Sulphate-reducing bacterial community structure from produced water of the Periquito and Galo de Campina onshore oilfields in Brazil. Sci. Rep..

[2] Pannekens M., Kroll L., Müller H., Mbow F.T., Meckenstock R.U. (2019). Oil reservoirs, an exceptional habitat for microorganisms. New Biotechnol..

[3] Liu J., Wu J., Lin J., Zhao J., Xu T., Yang Q., Zhao J., Zhao Z., Song X. (2019). Changes in the Microbial Community Diversity of Oil Exploitation. Genes.

[4] Yin J., Wei X., Hu F., Cheng C., Song M., Zhuang G., Ma A. (2023). Alternative stable microbiome state triggered by the introduction of functional microbes in oil reservoirs drives sustainable microbial enhanced oil recovery. Chem. Eng. J..

[5] Peng S., Li Z., Zhang D., Lu P., Zhou S. (2024). Changes in community structure and microbiological risks in a small stream after receiving treated shale gas wastewater for two years. Environ. Pollut..

[6] Pereira G.F., Pilz-Junior H.L., Corção G. (2021). The impact of bacterial diversity on resistance to biocides in oilfields. Sci. Rep..

[7] Kahrilas G.A., Blotevogel J., Stewart P.S., Borch T. (2015). Biocides in Hydraulic Fracturing Fluids: A Critical Review of Their Usage, Mobility, Degradation, and Toxicity. Environ. Sci. Technol..

[8] Campa M.F., Wolfe A.K., Techtmann S.M., Harik A.-M., Hazen T.C. (2019). Unconventional Oil and Gas Energy Systems: An Unidentified Hotspot of Antimicrobial Resistance?. Front. Microbiol..

[9] Chen P., Gao P., Chen Y., Xie J., Jin M., Ma T. (2020). Occurrence of antibiotic resistance genes in an oilfield’s water re-injection systems. Ecotoxicol. Environ. Saf..

[10] Alabbas F.M., Mishra B. (2016). Microbiologically Influenced Corrosion of Pipelines in the Oil & Gas Industry. Proceedings of the 8th Pacific Rim International Congress on Advanced Materials and Processing.

[11] https://www.reportsanddata.com/report-detail/corrosion-inhibitor-market.

[12] Cámara M., Green W., MacPhee C.E., Rakowska P.D., Raval R., Richardson M.C., Slater-Jefferies J., Steventon K., Webb J.S. (2022). Economic significance of biofilms: A multidisciplinary and cross-sectoral challenge. NPJ Biofilms Microbiomes.

[13] Askari M., Aliofkhazraei M., Jafari R., Hamghalam P., Hajizadeh A. (2021). Downhole corrosion inhibitors for oil and gas production—A review. Appl. Surf. Sci. Adv..

[14] Moore S.L., Cripps C.M. (2010). Bacterial Survival in Fractured Shale-Gas Wells of the Horn River Basin. J. Can. Pet. Technol..

[15] Daly R., Borton M., Wilkins M., Hoyt D.W., Kountz D.J., Wolfe R.A., Welch S.A., Marcus D.N., Trexler R.V., MacRae J.D. (2016). Microbial metabolisms in a 2.5-km-deep ecosystem created by hydraulic fracturing in shales. Nat. Microbiol..

[16] Arnaud C. (2016). Microbial communities thrive in fracking wells. C&EN Glob. Enterp..

[17] Barbot E., Vidic N., Gregory K.B., Vidic R.D. (2013). Spatial and temporal correlation of water quality parameters of produced waters from devonian-age shale following hydraulic fracturing. Environ. Sci. Technol..

[18] Eckert R.B. (2015). Emphasis on biofilms can improve mitigation of microbiologically influenced corrosion in oil and gas industry. Corros. Eng. Sci. Technol..

[19] Fichter J.K., Johnson K., French K., Oden R. Use of Microbiocides in Barnett Shale Gas Well Fracturing Fluids to Control Bacterially-Related Problems. Proceedings of the NACE International Corrosion Conference and Expo.

[20] Bottero S., Picioreanu C., Enzien M.V., Loosdrecht M.V., Bruining J., Heimovaara T. (2010). Formation Damage and Impact on Gas Flow Caused by Biofilms Growing Within Proppant Packing Used in Hydraulic Fracturing. SPE International Symposium and Exhibition on Formation Damage Control.

[21] Hull N.M., Rosenblum J.S., Robertson C.E., Harris J.K., Linden K.G. (2018). Succession of toxicity and microbiota in hydraulic fracturing flowback and produced water in the Denver–Julesburg Basin. Sci. Total Environ..

[22] Ulrich N., Kirchner V., Drucker R., Wright J.R., McLimans C.J., Hazen T.C., Campa M.F., Grant C.J., Lamendella R. (2018). Response of Aquatic Bacterial Communities to Hydraulic Fracturing in Northwestern Pennsylvania: A Five-Year Study. Sci. Rep..

[23] Mohan A.M., Hartsock A., Hammack R.W., Vidic R.D., Gregory K.B. (2013). Microbial communities in flowback water impoundments from hydraulic fracturing for recovery of shale gas. FEMS Microbiol. Ecol..

[24] Mohan A.M., Bibby K.J., Lipus D., Hammack R.W., Gregory K.B. (2014). The Functional Potential of Microbial Communities in Hydraulic Fracturing Source Water and Produced Water from Natural Gas Extraction Characterized by Metagenomic Sequencing. PLoS ONE..

[25] Strong L.C., Gould T., Kasinkas L., Sadowsky M.J., Aksan A., Wackett L.P. (2014). Biodegradation in Waters from Hydraulic Fracturing: Chemistry, Microbiology, and Engineering. J. Environ. Eng..

[26] Vikram A., Lipus D., Bibby K. (2014). Produced water exposure alters bacterial response to biocides. Environ. Sci. Technol..

[27] Vikram A., Lipus D., Bibby K. (2016). Metatranscriptome analysis of active microbial communities in produced water samples from the Marcellus Shale. Microbial. Ecol..

[28] Hunt S.M., Werner E.M., Huang B., Hamilton M.A., Stewart P.S. (2004). Hypothesis for the Role of Nutrient Starvation in Biofilm Detachment. Appl. Environ. Microbiol..

[29] Ghanbari A., Dehghany J., Schwebs T., Müsken M., Häussler S., Meyer-Hermann M. (2016). Inoculation density and nutrient level determine the formation of mushroom-shaped structures in *Pseudomonas aeruginosa* biofilms. Nat. Rev. Sci. Rep..

[30] Kahrilas G.A., Blotevogel J., Corrin E.R., Borch T. (2016). Downhole Transformation of the Hydraulic Fracturing Fluid Biocide Glutaraldehyde: Implications for Flowback and Produced Water Quality. Environ. Sci. Technol..

[31] Li Y., Jia R., Al-Mahamedh H.H., Xu D., Gu T. (2016). Enhanced Biocide Mitigation of Field Biofilm Consortia by a Mixture of D-Amino Acids. Front Microbiol..

[32] LaSarre B., Federle M.J. (2013). Exploiting quorum sensing to confuse bacterial pathogens. Microbiol. Mol. Biol. Rev..

[33] Shefner A.M., Burkhardt B.J. (1957). Reversal of boric acid inhibition of growth in certain soil microorganisms. Nature.

[34] Roush A., Norris E.R. (1950). The inhibition of xanthine oxidase by borates. Arch. Biochem..

[35] Ali S.E., Thoen E., Evensen Ø., Wiik-Nielsen J., Gamil A.A.A., Skaar I. (2014). Mitochondrial Dysfunction Is Involved in the Toxic Activity of Boric Acid against *Saprolegnia*. PLoS ONE.

[36] Houlsby R.D., Ghajar M., Chavez G.O. (1986). Antimicrobial activity of borate-buffered solutions. Antimicrob. Agents Chemother..

[37] Potos C. (1965). Effects of EDTA on Wastewater Treatment. J. Water Pollut. Control Fed..

[38] Smith B.L. (1990). Codex Alimentarius, Abridged Version.

[39] De Vuyst L., Vandamme E.J. (1994). Antimicrobial Potential of Lactic Acid Bacteria in Bacteriocins of Lactic Acid Bacteria.

[40] In Y.W., Kim J.J., Kim H.J., Oh S.W. (2013). Antimicrobial Activities of Acetic Acid, Citric Acid and Lactic Acid against Shigella Species. J. Food Saf..

[41] Phillips C.A. (1999). The effect of citric acid, lactic acid, sodium citrate and sodium lactate, alone and in combination with nisin, on the growth of *Arcobacter butzleri*. Lett. Appl. Microbiol..

[42] Cowan M.M. (1999). Plant products as antimicrobial agents. Clin. Microbiol. Rev..

[43] Serafini M., Ghiselli A., Ferro-Luzzi A. (1994). Red wine, tea and anti-oxidants. Lancet.

[44] Chung K.T., Lu Z., Chou M.W. (1998). Mechanism of inhibition of tannic acid and related compounds on the growth of intestinal bacteria. Food Chem. Toxicol..

[45] Logan J. (1989). Tannic Acid Treatment.

[46] Çolak S.M., Yapici B.M., Yapici A.N. (2010). Determination of antimicrobial activity of tannic acid in pickling process. Rom. Biotechnol. Lett..

[47] Scalbert A. (1991). Antimicrobial properties of tannins. Phytochemistry.

[48] Ya C., Gaffney S.H., Lilley T.H., Haslam E., Hemingway R.W., Karchesy J.J. (1988). Carbohydratepolyphenol complexation. Chemistry and Significance of Condensed Tannins.

[49] Seminara A., Angelini T.E., Wilking J.N., Vlamakis H., Ebrahim S., Kolter R., Weitz D.A., Brenner M.P. (2012). Osmotic spreading of *Bacillus subtilis* biofilms driven by an extracellular matrix. Proc. Natl. Acad. Sci. USA.

[50] Farrell F.D.C., Hallatschek O., Marenduzzo D., Waclaw B. (2013). Mechanically driven growth of quasi-two-dimensional microbial colonies. Phys. Rev. Lett..

[51] De Seta F., Schmidt M., Vu B., Essmann M., Larsen B. (2009). Antifungal mechanisms supporting boric acid therapy of *Candida vaginitis*. J. Antimicrob. Chemother..

[52] Reichman O., Akins R., Sobel J.D. (2009). Boric acid addition to suppressive antimicrobial therapy for recurrent bacterial vaginosis. Sex Transm Dis..

[53] Banin E., Brady K.M., Greenberg E.P. (2006). Chelator-induced dispersal and killing of *Pseudomonas aeruginosa* cells in a biofilm. Appl. Environ. Microbiol..

[54] Payne D.E., Martin N.R., Parzych K.R., Rickard A.H., Underwood A., Boles B.R. (2013). Tannic acid inhibits *Staphylococcus aureus* surface colonization in an IsaA-dependent manner. Infect. Immun..

[55] Sambrook J., Fritsch E.F., Maniatis T. (1989). Molecular Cloning: A Laboratory Manual.

[56] King M.D., McFarland A.R. (2012). Bioaerosol Sampling with a Wetted Wall Cyclone: Cell Culturability and DNA Integrity of *Escherichia coli* Bacteria. Aerosol Sci. Technol..

[57] (1995). Standard Method 2540C (adapted from ASTM D2974). https://uwlab.soils.wisc.edu/wp-content/uploads/sites/17/2015/08/DNR_Total_Solids.pdf.

[58] Olson W.P. (1996). Automated Microbial Identification and Quantitation.

[59] Zhou C., Yang Y., Jong A.Y. (1990). Mini-prep in ten minutes. Biotechniques.

[60] Estrada-Perez C.E., Kinney K.A., Maestre J.P., Hassan Y.A., King M.D. (2018). Droplet Distribution and Airborne Bacteria in an Experimental Shower Unit. Water Res..

[61] Wilson C., Lukowicz R., Merchant S., Valquier-Flynn H., Caballero J., Sandoval J., Okuom M., Huber C., Brooks T.D., Wilson E. (2017). Quantitative and Qualitative Assessment Methods for Biofilm Growth: A Mini-review. Res. Rev. J. Eng. Technol..

[62] Bakkiyaraj D., Pandian S.T.K. (2010). In vitro and in vivo antibiofilm activity of a coral associated actinomycete against drug resistant *Staphylococcus aureus* biofilms. Biofouling J. Bioadhesion Biofilm Res..

[63] Merritt J.H., Kadouri D.E., O’Toole G.A. (2011). Growing and analyzing static biofilms. Curr. Protoc. Microbiol..

[64] Baldassarri L., Creti R., Recchia S., Pataracchia M., Alfarone G., Orefici G., Campoccia D., Montanaro L., Arciola C.R. (2006). Virulence factors in enterococcal infections of orthopedic devices. Int. J. Artif. Organs..

[65] Frenkiel-Krispin D., Sack R., Englander J., Shimoni E., Eisenstein M., Bullitt E., Horowitz-Scherer R., Hayes C.S., Setlow P., Minsky A. (2004). Structure of the DNA-SspC Complex: Implications for DNA Packaging, Protection, and Repair in Bacterial Spores. J. Bacteriol..

[66] Rode L.J., Lewis C.W., Foster J.W. (1962). Electron microscopy of spores of *Bacillus megaterium* with special reference to the effects of fixation and thin sectioning. J. Cell Biol..

[67] Gerhardt P., Black S.H. (1961). Permeability of bacterial spores. II. Molecular variables affecting solute permeation. J. Bacteriol..

[68] Setlow P. (2013). When the sleepers wake: The germination of spores of *Bacillus* species. J. Appl. Microbiol..

[69] Swerdlow B.M., Setlow B., Setlow P. (1981). Levels of H^+^ and other monovalent cations in dormant and germinated spores of *Bacillus megaterium*. J. Bacteriol..

[70] Peng L., Chen D., Setlow P., Li Y.-Q. (2009). Elastic and inelastic light scattering from single bacterial spores in an optical trap allows the monitoring of spore germination dynamics. Anal. Chem..

[71] Lund P.A., De Biase D., Liran O., Scheler O., Mira N.P., Cetecioglu Z., Fernández E.N., Bover-Cid S., Hall R., Sauer M. (2020). Understanding How Microorganisms Respond to Acid pH Is Central to Their Control and Successful Exploitation. Front. Microbiol..

[72] Wade W. (2002). Unculturable bacteria—The uncharacterized organisms that cause oral infections. J. R. Soc. Med..

[73] Knisely R.F. (1966). Selective medium for *Bacillus anthracis*. J. Bacteriol..

