# Supplementary material for: Inhibition of Microbial Growth and Biofilm Formation in Pure and Mixed Bacterial Samples
Source: Microorganisms. 2024 Jul 22;12(7):1500. doi: 10.3390/microorganisms12071500 (PMC11278618; doi:10.3390/microorganisms12071500)
Supplement: Supplementary file 1 [file microorganisms-12-01500-s001.zip › microorganisms-3064487-supplementary.pdf]

Supporting Information

Table S1. Classification of the genera delineated in the fracturing water microbiome.

Bacteria	Gram-negative	% Total	Gram-positive	% Total
Non-Spore Forming	40	60	7	10
Spore Forming	9	13.5	5	7.5
Unknown	5	7.5	1	1.5
Total Bacteria	54 (of 67 Total)	81	13 (of 67 Total)	19

Table S2a

Table S2.a. Absorbance values for the bacterial liquid cultures at different dilutions and biocide concentrations.

				BG Liquid Culture				
**Boric acid**	**10^7/mL**	**STDEV**	**10^6/mL**	**STDEV**	**10^5/mL**	**STDEV**	**10^4/mL**	**STDEV**
**0 g/L**	1	0.099177	1	0.067146	1	0.044583	1	0.070904
**100 mg/L**	0.87025	0.06761	0.77792	0.033431	0.787593	0.039387	0.834771	0.027385
**250 mg/L**	0.949698	0.090437	0.829318	0.034659	0.849561	0.004102	0.848714	0.039661
**500 mg/L**	0.945033	0.082846	0.909454	0.014901	0.904877	0.04329	0.912403	0.049175
**1 g/L**	0.916802	0.106878	0.98373	0.064558	0.912494	0.052995	0.92155	0.053525
**5 g/L**	0.371505	0.014303	0.575102	0.022662	0.652593	0.010479	0.695717	0.030874
**EDTA**	**10^7/mL**	**STDEV**	**10^6/mL**	**STDEV**	**10^5/mL**	**STDEV**	**10^4/mL**	**STDEV**
**0 g/L**	1	0.137554	1	0.051403	1	0.050196	1	0.030671
**100 mg/L**	0.71517	0.066214	0.6613	0.012626	0.78957	0.004257	0.79639	0.014949
**250 mg/L**	0.682366	0.022265	0.667875	0.02945	0.830555	0.072775	0.791715	0.0115
**500 mg/L**	0.533789	0.020564	0.594125	0.009905	0.825845	0.073811	0.834522	0.015647
**1 g/L**	0.330934	0.094834	0.1825	0.007366	0.212051	0.002571	0.197275	0.020095
**5 g/L**	0.153126	0.017682	0.17635	0.005923	0.214651	0.006153	0.196691	0.022054
**Lactic acid**	**10^7/mL**	**STDEV**	**10^6/mL**	**STDEV**	**10^5/mL**	**STDEV**	**10^4/mL**	**STDEV**
**0 g/L**	1	0.05149	1	0.092133	1	0.040746	1	0.016919
**100 mg/L**	0.914326	0.016051	0.741886	0.00982	0.74864	0.039914	0.817108	0.04968
**250 mg/L**	0.951409	0.056164	0.695432	0.015092	0.797509	0.02733	0.82361	0.037358
**500 mg/L**	0.950567	0.01331	0.748497	0.028588	0.804764	0.02087	0.81869	0.067186
**1 g/L**	0.968495	0.054487	0.781101	0.080356	0.828172	0.044808	0.817108	0.044834
**5 g/L**	0.791201	0.039467	0.745476	0.01238	0.832655	0.004735	0.791453	0.109027
**Tannic acid**	**10^7/mL**	**STDEV**	**10^6/mL**	**STDEV**	**10^5/mL**	**STDEV**	**10^4/mL**	**STDEV**
**0 g/L**	1	0.077673	1	0.049186	1	0.026534	1	0.057637
**100 mg/L**	0.735601	0.041032	0.919095	0.096527	0.732852	0.010175	0.804791	0.035261
**250 mg/L**	0.631727	0.04082	1.01271	0.041708	0.695572	0.031142	0.681352	0.046398
**500 mg/L**	0.465533	0.051464	0.634837	0.024307	0.644318	0.010168	0.622741	0.015578
**1 g/L**	0.382714	0.011563	0.715657	0.041352	0.505895	0.170448	0.308702	0.013058
**5 g/L**	0.169332	0.013021	0.281851	0.039944	0.314915	0.002466	0.428078	0.012495
				**E. coli Liquid Culture**				
**Boric acid**	**10^7/mL**	**STDEV**	**10^6/mL**	**STDEV**	**10^5/mL**	**STDEV**	**10^4/mL**	**STDEV**
**0 g/L**	1	0.021128	1	0.061229	1	0.089756	1	0.032327
**100 mg/L**	0.924824	0.045092	0.873343	0.010594	0.917722	0.105835	0.934539	0.063375
**250 mg/L**	0.961739	0.063003	0.920984	0.037235	0.942791	0.042647	1.000327	0.027641
**500 mg/L**	0.910853	0.055921	0.911833	0.035461	0.96131	0.046338	0.972298	0.022882
**1 g/L**	0.899007	0.021113	0.910594	0.005925	0.928329	0.052673	0.977892	0.040217
**5 g/L**	0.886801	0.041408	0.904631	0.029664	0.917461	0.004477	0.977208	0.042556
**EDTA**	**10^7/mL**	**STDEV**	**10^6/mL**	**STDEV**	**10^5/mL**	**STDEV**	**10^4/mL**	**STDEV**
**0 g/L**	1	0.055292	1	0.036235	1	0.06221	1	0.033258
**100 mg/L**	0.838496	0.009223	0.826758	0.008391	0.768255	0.012698	0.857155	0.018509
**250 mg/L**	0.891915	0.058303	0.882676	0.009427	0.809559	0.016659	0.865737	0.019067
**500 mg/L**	0.899802	0.030464	0.897397	0.056851	0.777241	0.043724	0.94004	0.051612

**1 g/L**	0.813752	0.008979	0.800671	0.040584	0.787251	0.018218	0.850128	0.022696
**5 g/L**	0.700816	0.010808	0.745169	0.021845	0.734307	0.02982	0.80883	0.035143
**Lactic acid**	**10^7/mL**	**STDEV**	**10^6/mL**	**STDEV**	**10^5/mL**	**STDEV**	**10^4/mL**	**STDEV**
**0 g/L**	1	0.088683	1	0.036544	1	0.023007	1	0.065177
**100 mg/L**	1.038784	0.129678	0.918636	0.01921	0.917382	0.078907	0.99004	0.026787
**250 mg/L**	0.999301	0.027587	0.962385	0.010429	0.832192	0.025796	0.919431	0.089412
**500 mg/L**	1.005667	0.011842	0.882809	0.02656	0.838237	0.070646	0.80773	0.017501
**1 g/L**	1.038453	0.033466	0.967258	0.033187	0.865086	0.064945	0.869072	0.031736
**5 g/L**	0.998105	0.012088	0.908627	0.040608	0.875088	0.037024	0.889701	0.033744
**Tannic acid**	**10^7/mL**	**STDEV**	**10^6/mL**	**STDEV**	**10^5/mL**	**STDEV**	**10^4/mL**	**STDEV**
**0 g/L**	1	0.021462	1	0.034799	1	0.027238	1	0.061004
**100 mg/L**	0.932311	0.165901	0.841789	0.040633	0.820451	0.016743	0.830613	0.032117
**250 mg/L**	0.727987	0.036571	0.791178	0.013846	0.756426	0.005052	0.782127	0.009708
**500 mg/L**	0.74385	0.086473	0.762683	0.019897	0.713249	0.01219	0.765386	0.036078
**1 g/L**	0.751154	0.050724	0.747585	0.037273	0.752428	0.013081	0.756371	0.061288
**5 g/L**	0.597838	0.032633	0.601707	0.031119	0.630419	0.060248	0.638855	0.104062
				**Fracturing Water Liquid Culture**				
**Boric acid**	**10^7/mL**	**STDEV**	**10^6/mL**	**STDEV**	**10^5/mL**	**STDEV**	**10^4/mL**	**STDEV**
**0 g/L**	1	0.139851	1	0.570557	1	0.575045	1	0.023625
**100 mg/L**	0.699312	0.204925	0.991086	0.769262	0.24718	0.000436	0.781481	0.001039
**250 mg/L**	0.901616	0.328686	0.289909	0.002419	0.830805	0.443507	0.985185	0.026001
**500 mg/L**	1.009339	0.1665	1.138196	0.847406	0.325749	0.003219	0.920782	0.017307
**1 g/L**	0.947321	0.082283	0.289618	0.002722	0.33463	0.004876	0.970165	0.022073
**5 g/L**	0.722448	0.142806	0.914822	0.612112	0.327823	0.003745	0.94465	0.020085
**EDTA**	**10^7/mL**	**STDEV**	**10^6/mL**	**STDEV**	**10^5/mL**	**STDEV**	**10^4/mL**	**STDEV**
**0 g/L**	1	0.176823	1	0.007988	1	0.386695	1	0.022806
**100 mg/L**	0.815967	0.087199	1.436531	0.2388	0.717947	0.270902	0.808647	0.002401
**250 mg/L**	0.89772	0.137093	3.40696	0.459755	0.424816	0.002406	0.901755	0.013204
**500 mg/L**	0.821578	0.34176	2.615759	0.539996	0.423411	0.002859	0.921661	0.015611
**1 g/L**	0.828913	0.169143	0.932023	0.009364	0.437546	0.002603	0.975599	0.022407
**5 g/L**	0.122725	0.026594	0.936351	0.002951	0.437216	0.008402	0.997003	0.016266
**Lactic acid**	**10^7/mL**	**STDEV**	**10^6/mL**	**STDEV**	**10^5/mL**	**STDEV**	**10^4/mL**	**STDEV**
**0 g/L**	1	0.109642	1	0.418667	1	0.006848	1	0.017229
**100 mg/L**	0.869032	0.250561	0.991872	0.505122	0.839626	0.002425	0.861349	0.008879
**250 mg/L**	0.847949	0.141126	1.02382	0.449068	0.958034	0.002524	0.948684	0.008199
**500 mg/L**	0.908471	0.173625	0.449392	0.003765	0.929017	0.004279	0.916776	0.01369
**1 g/L**	0.892683	0.71733	0.440557	0.004532	0.963027	0.004966	0.968586	0.009057
**5 g/L**	0.363198	0.28887	0.650905	0.178473	0.931981	0.007165	0.939967	0.007114
**Tannic acid**	**10^7/mL**	**STDEV**	**10^6/mL**	**STDEV**	**10^5/mL**	**STDEV**	**10^4/mL**	**STDEV**
**0 g/L**	1	0.228952	1	0.460064	1	0.000889	1	0.012011
**100 mg/L**	0.985515	0.275855	0.441268	0.007703	1.009264	0.006461	1.042321	0.019032
**250 mg/L**	0.652217	0.188908	0.660563	0.067223	2.344345	0.345315	1.32125	0.006358
**500 mg/L**	0.45089	0.207618	0.619744	0.002875	1.495193	0.004341	1.46375	0.011092
**1 g/L**	0.381248	0.025232	0.753782	0.01117	1.787275	0.00542	1.736429	0.006806
**5 g/L**	0.303793	0.016877	0.81986	0.056291	1.604964	0.029905	1.819643	0.0269

Table S2. b

Table S2b. Absorbance values for bacterial biofilms at different dilutions and biocide concentrations.

				**BG Biofilm**				
**Boric acid**	**10^7/mL**	**STDEV**	**10^6/mL**	**STDEV**	**10^5/mL**	**STDEV**	**10^4/mL**	**STDEV**
**0 g/L**	1	0.047604	1	0.276341	1	0.431664	1	0.137193
**100 mg/L**	1.002609	0.01908	1.018605	0.102942	1.107388	0.126402	1.110406	0.003814
**250 mg/L**	1.011306	0.042407	0.811489	0.257727	0.935849	0.168992	0.949587	0.292658
**500 mg/L**	1.003354	0.061481	1.095156	0.021968	1.175932	0.075875	0.97344	0.142745

**1 g/L**	0.979639	0.02861	1.075783	0.095901	1.196623	0.023042	0.907407	0.306366
**5 g/L**	0.766972	0.309529	1.05035	0.088302	1.201256	0.005302	0.928185	0.440915
**EDTA**	**10^7/mL**	**STDEV**	**10^6/mL**	**STDEV**	**10^5/mL**	**STDEV**	**10^4/mL**	**STDEV**
**0 g/L**	1	0.072819	1	0.027573	1	0.014174	1	0.019135
**100 mg/L**	10.00865	0.028162	18.26242	0.031449	19.99856	0.065462	17.46811	0.020665
**250 mg/L**	3.962254	0.025047	7.424955	0.011857	8.212738	0.026117	7.553994	0.105342
**500 mg/L**	3.92233	0.095862	7.551963	0.016483	8.427281	0.020394	7.337801	0.230478
**1 g/L**	3.879725	0.057676	7.721519	0.058239	8.553817	0.012758	6.68846	0.09192
**5 g/L**	3.990005	0.271989	1.261568	0.035867	0.962557	0.030456	0.957502	0.04888
**Lactic acid**	**10^7/mL**	**STDEV**	**10^6/mL**	**STDEV**	**10^5/mL**	**STDEV**	**10^4/mL**	**STDEV**
**0 g/L**	1	0.125688	1	0.033889	1	0.019415	1	0.167423
**100 mg/L**	0.93492	0.009753	0.994839	0.010294	0.992609	0.060367	1.113839	0.04062
**250 mg/L**	0.943117	0.024051	0.853123	0.297169	0.90241	0.256808	1.010932	0.308026
**500 mg/L**	0.932914	0.076449	0.99206	0.014614	0.999002	0.017197	0.949065	0.28609
**1 g/L**	0.941911	0.017379	0.998046	0.004583	0.99706	0.022285	1.067804	0.03822
**5 g/L**	0.927179	0.025546	0.99517	0.009494	0.999764	0.016702	1.052914	0.153809
**Tannic acid**	**10^7/mL**	**STDEV**	**10^6/mL**	**STDEV**	**10^5/mL**	**STDEV**	**10^4/mL**	**STDEV**
**0 g/L**	1	0.022774	1	0.008147	1	0.005254	1	0.025571
**100 mg/L**	0.908771	0.016456	0.986102	0.0051	0.995477	0.029673	0.998803	0.005823
**250 mg/L**	0.925909	0.038784	1.006155	0.01022	1.000112	0.017328	1.017162	0.034698
**500 mg/L**	0.911621	0.036945	1.007181	0.019881	0.999874	0.009582	1.001682	0.003831
**1 g/L**	0.90162	0.02155	1.002825	0.018023	0.772381	0.924173	0.494206	0.787956
**5 g/L**	0.599723	0.149141	0.84036	0.448825	1.022672	0.031568	0.968228	0.179039
**E. coli Biofilm**								
**Boric acid**	**10^7/mL**	**STDEV**	**10^6/mL**	**STDEV**	**10^5/mL**	**STDEV**	**10^4/mL**	**STDEV**
**0 g/L**	1	0.003338	1	0.007544	1	0.003758	1	0.013372
**100 mg/L**	0.991785	0.00611	0.984753	0.003362	0.986135	0.006396	0.998085	0.035127
**250 mg/L**	1.000157	0.023044	1.00586	0.021508	0.997016	0.002797	0.995828	0.012344
**500 mg/L**	1.002378	0.00418	0.997582	0.007158	1.00217	0.008697	0.996699	0.006915
**1 g/L**	1.003374	0.003525	0.998933	0.00151	0.999443	0.006096	1.000157	0.011279
**5 g/L**	1.001438	0.020471	1.006372	0.016438	1.004727	0.005581	0.9983	0.005255
**EDTA**	**10^7/mL**	**STDEV**	**10^6/mL**	**STDEV**	**10^5/mL**	**STDEV**	**10^4/mL**	**STDEV**
**0 g/L**	1	0.012279	1	0.009802	1	0.015215	1	0.008472
**100 mg/L**	0.985003	0.014627	0.985717	0.006596	0.974187	0.009378	0.9871	0.026885
**250 mg/L**	0.996798	0.01937	0.996958	0.009417	0.990376	0.006255	0.997576	0.011694
**500 mg/L**	1.009338	0.025441	1.009629	0.015096	0.993924	0.018429	0.996073	0.041635
**1 g/L**	0.995085	0.011317	0.994828	0.007674	0.991046	0.014809	1.006478	0.070187
**5 g/L**	0.995872	0.014362	0.99871	0.010458	0.995614	0.009297	1.012049	0.060651
**Lactic acid**	**10^7/mL**	**STDEV**	**10^6/mL**	**STDEV**	**10^5/mL**	**STDEV**	**10^4/mL**	**STDEV**
**0 g/L**	1	0.031778	1	0.018113	1	0.008599	1	0.037164
**100 mg/L**	0.980114	0.030934	0.978789	0.011643	0.971945	0.016249	0.979555	0.013203
**250 mg/L**	0.998221	0.012911	1.000014	0.010324	0.999986	0.003308	0.997782	0.035133
**500 mg/L**	0.997531	0.037511	1.003129	0.007315	1.004587	0.002138	0.997006	0.015924
**1 g/L**	0.996359	0.034424	1.009882	0.009789	1.003255	0.005853	1.004851	0.049557
**5 g/L**	0.989754	0.022043	1.002881	0.011801	1.002458	0.012795	0.999071	0.032846
**Tannic acid**	**10^7/mL**	**STDEV**	**10^6/mL**	**STDEV**	**10^5/mL**	**STDEV**	**10^4/mL**	**STDEV**
**0 g/L**	1	0.029151	1	0.01885	1	0.006897	1	0.036074
**100 mg/L**	0.983585	0.008723	0.974584	0.006486	0.970392	0.004004	0.974057	0.008514
**250 mg/L**	0.957549	0.108072	1.012129	0.010825	1.005963	0.014174	0.882555	0.343183
**500 mg/L**	0.929248	0.290621	1.013844	0.012056	1.021245	0.041586	0.821978	0.388277
**1 g/L**	0.918642	0.158531	0.996194	0.005519	0.995812	0.015168	0.913116	0.219422
**5 g/L**	0.996413	0.017936	1.067242	0.096908	1.078158	0.032871	1.049466	0.097682
**Fracturing Water Biofilm**								
**Boric acid**	**10^7/mL**	**STDEV**	**10^6/mL**	**STDEV**	**10^5/mL**	**STDEV**	**10^4/mL**	**STDEV**
**0 g/L**	1	0.210817	1	0.070437	1	0.06507	1	0.015053
**100 mg/L**	0.855964	0.180951	0.852082	0.01513	0.902678	0.019352	0.984809	0.029752

**250 mg/L**	0.421375	0.073176	0.801819	0.020162	0.807598	0.004879	0.933964	0.016357
**500 mg/L**	0.576144	0.164833	1.00742	0.096661	0.883105	0.020247	1.027748	0.027321
**1 g/L**	0.717814	0.196413	1.366084	0.28023	0.871959	0.012606	0.923733	0.010728
**5 g/L**	0.537077	0.176712	1.281355	0.261109	0.838657	0.005452	0.937219	0.015938
**EDTA**	**10^7/mL**	**STDEV**	**10^6/mL**	**STDEV**	**10^5/mL**	**STDEV**	**10^4/mL**	**STDEV**
**0 g/L**	1	0.231158	1	0.004706	1	0.369842	1	0.016501
**100 mg/L**	0.916078	0.345587	0.862117	0.006088	0.464534	0.012816	0.912881	0.025753
**250 mg/L**	1.366677	0.420158	2.996096	0.486942	0.541787	0.023403	0.982413	0.030389
**500 mg/L**	0.722062	0.058493	1.189725	0.051055	0.510105	0.008952	1.115291	0.025321
**1 g/L**	0.612128	0.116591	1.108682	0.012638	0.558096	0.019695	1.106497	0.011915
**5 g/L**	0.355384	0.010516	1.113367	0.016951	0.544518	0.019463	1.105683	0.011573
**Lactic acid**	**10^7/mL**	**STDEV**	**10^6/mL**	**STDEV**	**10^5/mL**	**STDEV**	**10^4/mL**	**STDEV**
**0 g/L**	1	0.050763	1	0.131484	1	0.006951	1	0.010821
**100 mg/L**	1.209065	0.070867	0.643571	0.084064	1.394213	0.086286	1.00914	0.00446
**250 mg/L**	1.518193	0.150279	0.749064	0.115874	0.948292	0.002684	1.040761	0.006661
**500 mg/L**	1.195021	0.04249	0.470662	0.014232	0.974383	0.004202	1.042984	0.006408
**1 g/L**	0.594319	0.544005	0.503745	0.00756	1.016603	0.011254	1.076087	0.006065
**5 g/L**	0.861315	0.103915	0.479775	0.015698	1.092979	0.0104	1.194417	0.009701
**Tannic acid**	**10^7/mL**	**STDEV**	**10^6/mL**	**STDEV**	**10^5/mL**	**STDEV**	**10^4/mL**	**STDEV**
**0 g/L**	1	0.170342	1	0.542422	1	0.024014	1	0.008195
**100 mg/L**	1.083677	0.4278	0.30624	0.016267	1.082533	0.01347	1.153732	0.002869
**250 mg/L**	0.891866	0.172586	0.712267	0.343067	3.397373	0.675452	1.214039	0.011125
**500 mg/L**	0.59934	0.100656	0.395963	0.018583	1.644972	0.037152	1.532872	0.008208
**1 g/L**	0.58615	0.01277	0.448785	0.001646	1.596942	0.031122	1.74518	0.022084
**5 g/L**	0.839929	0.04016	0.921734	0.053893	2.585964	0.0133	3.107266	0.043432

Figures S1-S14

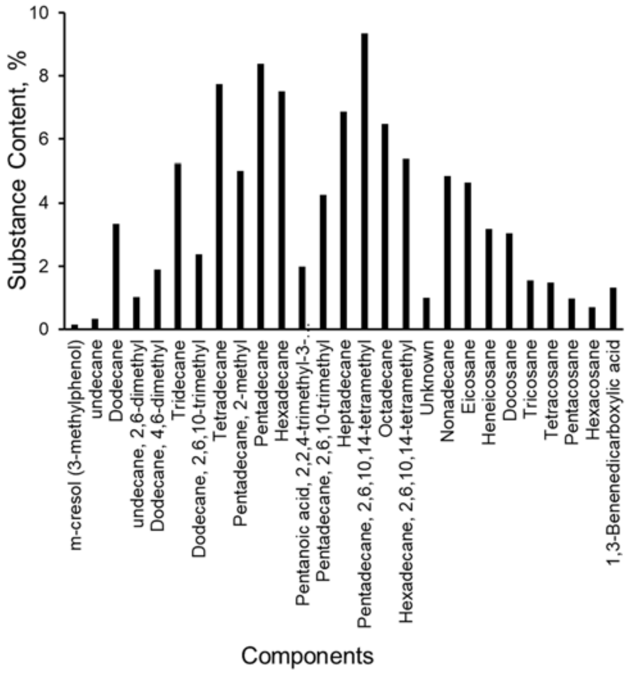

Figure S1. GC-MS analysis of fracturing water sample.

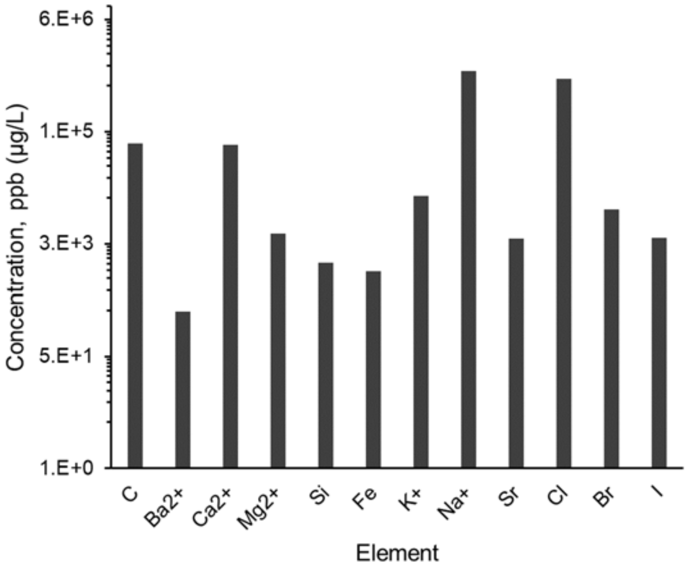

Figure S2. Aqueous elemental composition of the back-produced fracturing water collected at Ford Shale, TX. All concentrations are reported in µg/L (ppb).

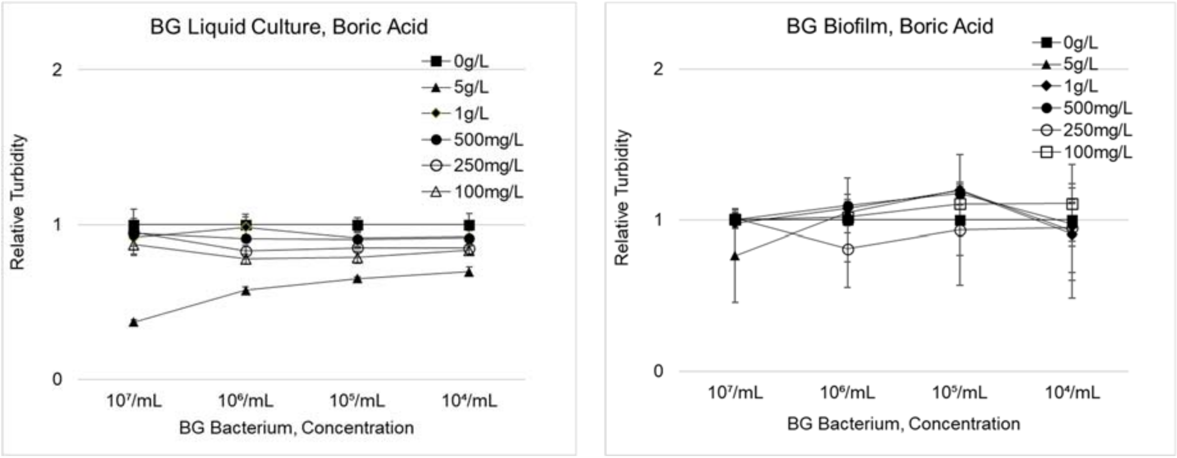

(a) **(b)**

Figure S3. (**a**) The effect of increasing boric acid concentrations on the growth of BG liquid cultures and (**b**) on the formation of BG biofilms.

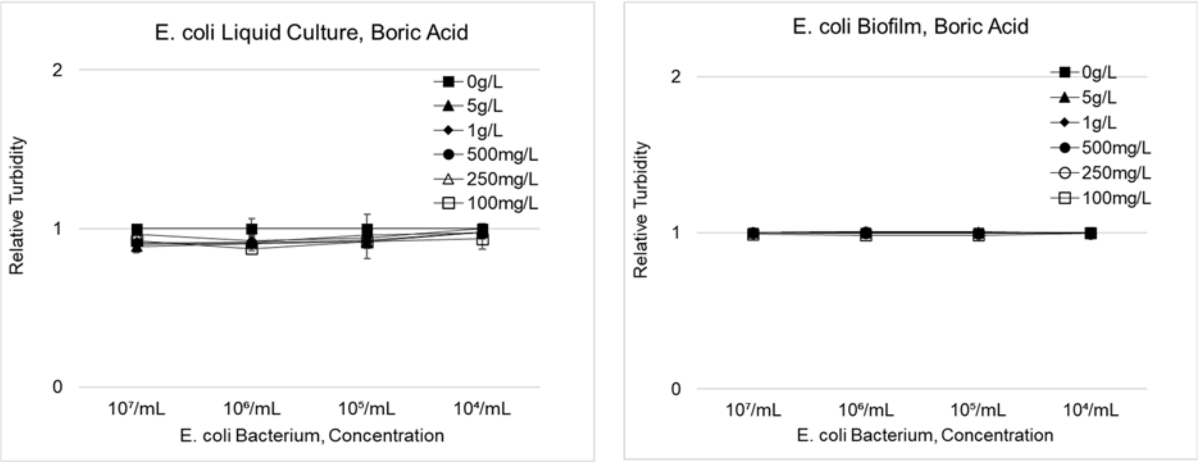

(a) **(b)**

Figure S4. (**a**). The effect of increasing boric acid concentrations on the growth of *E. coli* liquid cultures and (**b**) on the formation of *E. coli* biofilms.

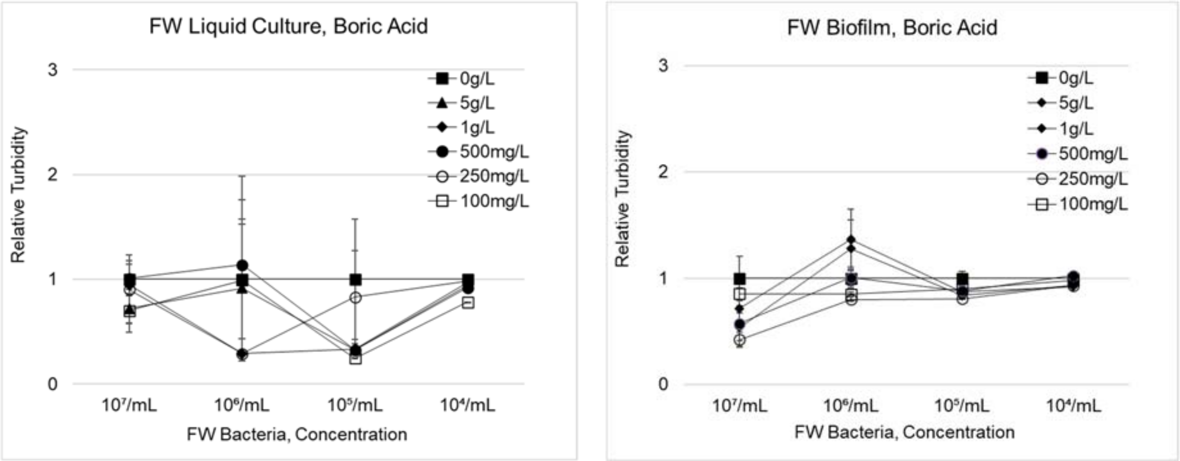

(a) **(b)**

Figure S5. (**a**) The effect of increasing boric acid concentrations on the growth of FW liquid cultures and (**b**) on the formation of FW biofilms.

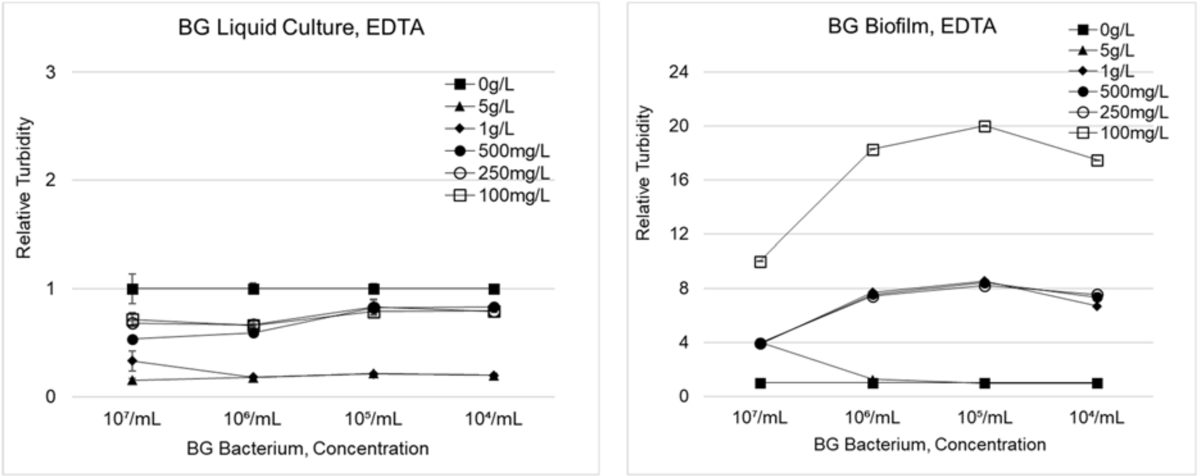

(a) **(b)**

Figure S6. (**a**) The effect of increasing EDTA concentrations on the growth of BG liquid cultures and (**b**) on the formation of BG biofilms.

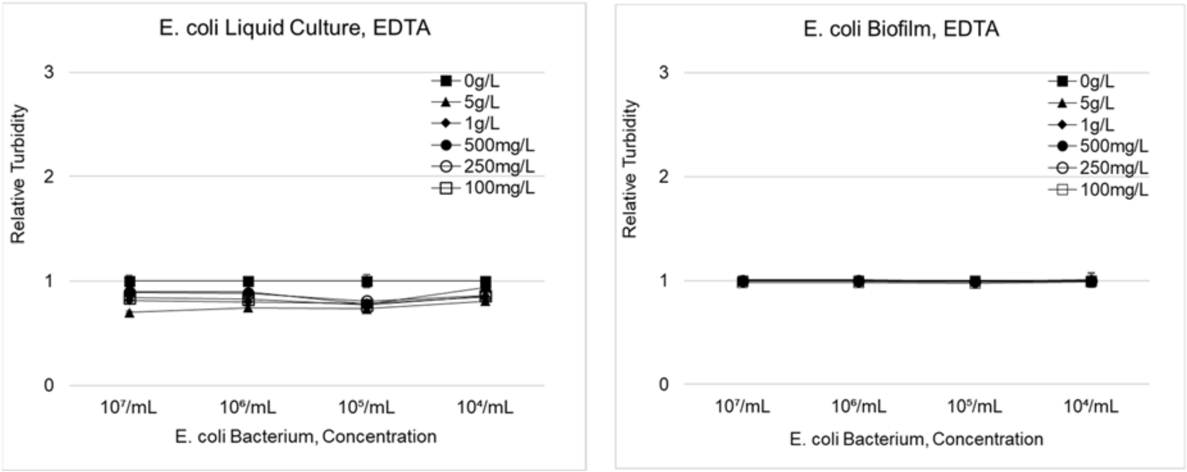

(a) **(b)**

Figure S7. (**a**). The effect of increasing EDTA concentrations on the growth of *E. coli* liquid cultures and (**b**) on the formation of *E. coli* biofilms.

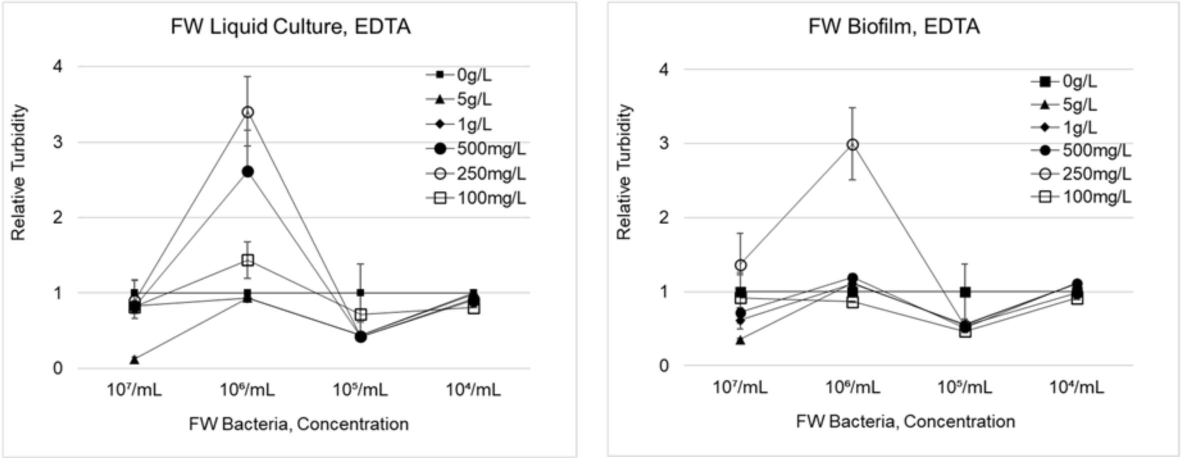

(a) **(b)**

Figure S8. (**a**) The effect of EDTA solutions on the growth of back-produced fracturing water (FW) liquid cultures and (**b**) on the formation of FW biofilm.

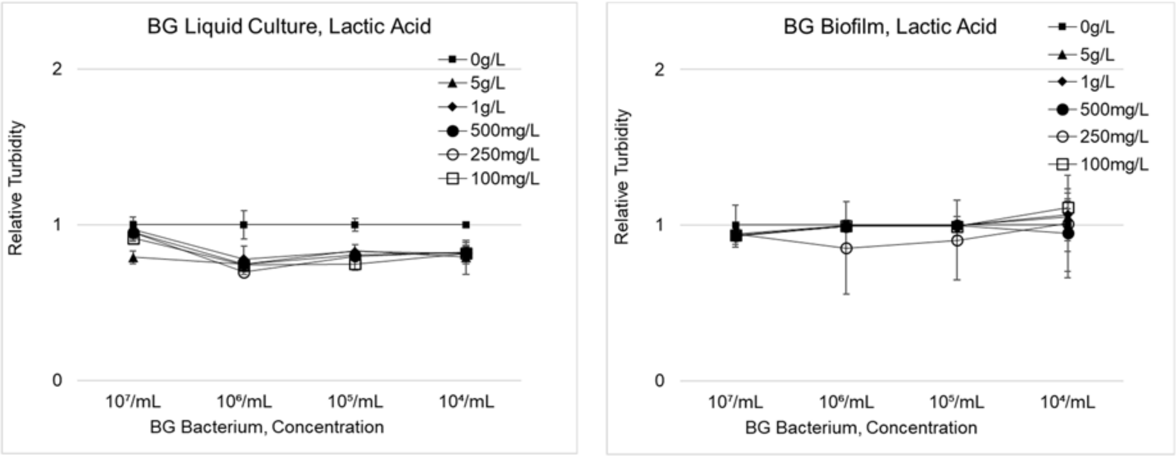

(a) **(b)**

Figure S9. (**a**) The effect of increasing lactic acid concentrations on the growth of BG liquid cultures and (**b**) on the formation of BG biofilms.

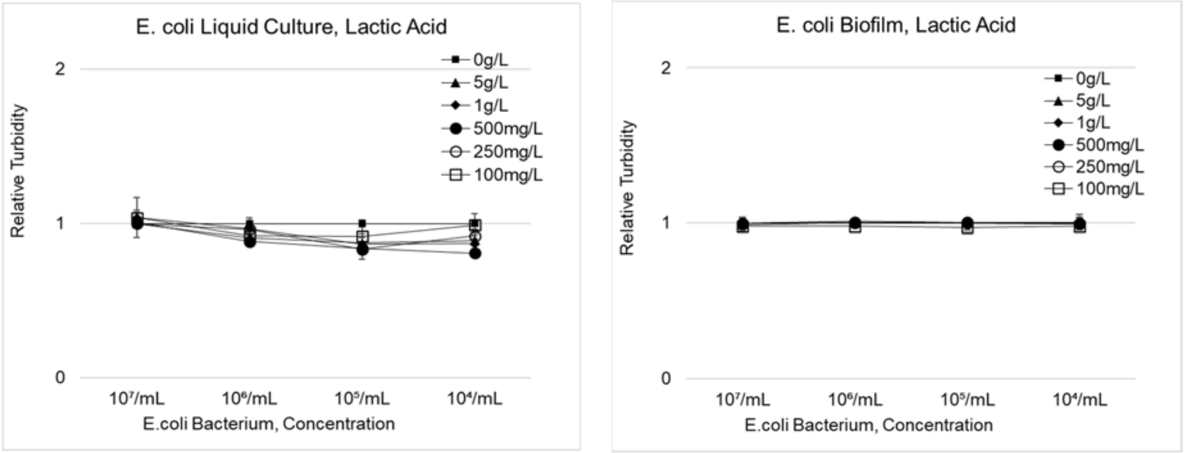

(a) **(b)**

Figure S10. (**a**) The effect of increasing lactic acid concentrations on the growth of *E. coli* liquid cultures and (**b**) on the formation of *E. coli* biofilms.

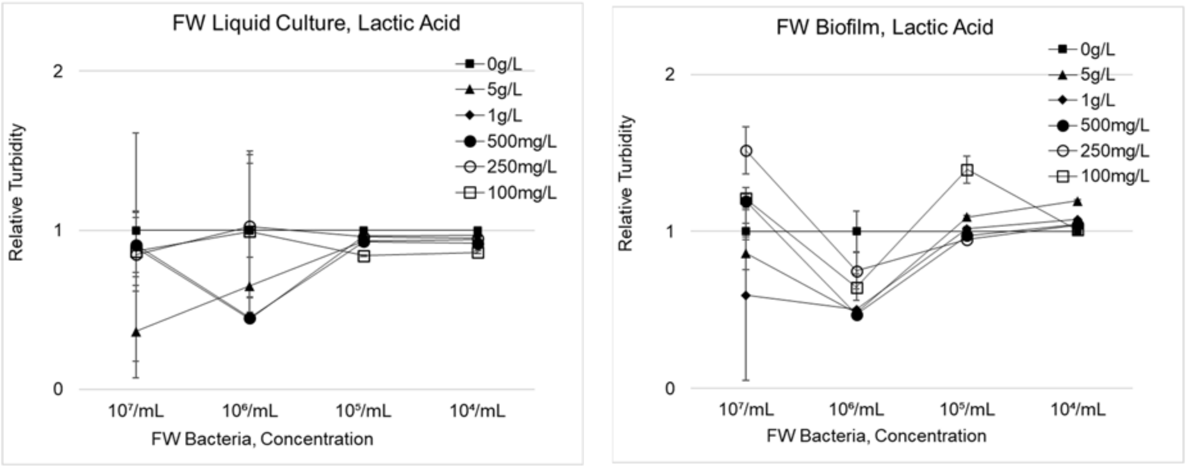

(a) **(b)**

Figure S11. (**a**) The effect of increasing lactic acid concentrations on the growth of FW liquid cultures and (**b**) on the formation of FW biofilms.

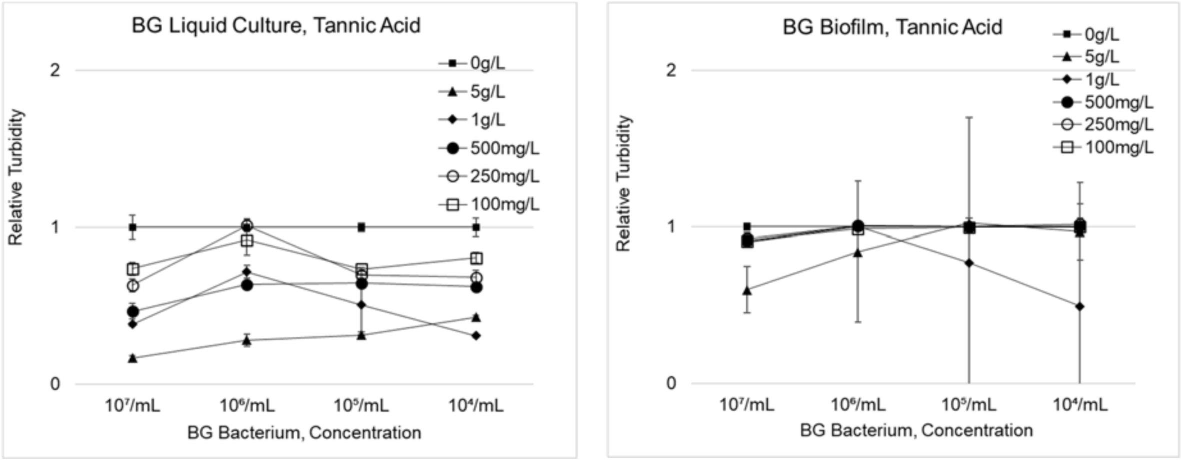

(a) **(b)**

Figure S12. (**a**) The effect of increasing tannic acid concentrations on the growth of BG liquid cultures and (**b**) on the formation of BG biofilms.

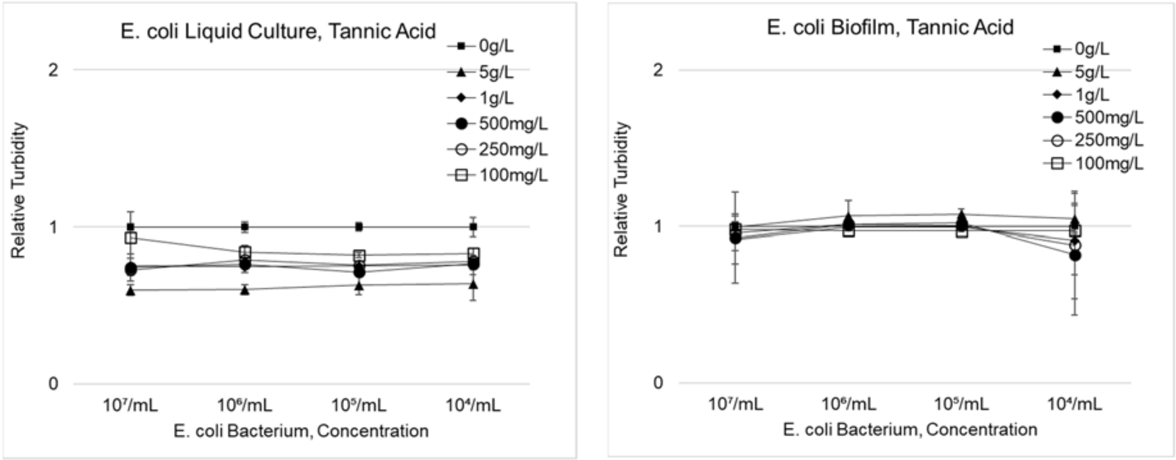

(a) **(b)**

Figure S13. (**a**) The effect of increasing tannic acid concentrations on the growth of *E. coli* liquid cultures and (**b**) on the formation of *E. coli* biofilms.

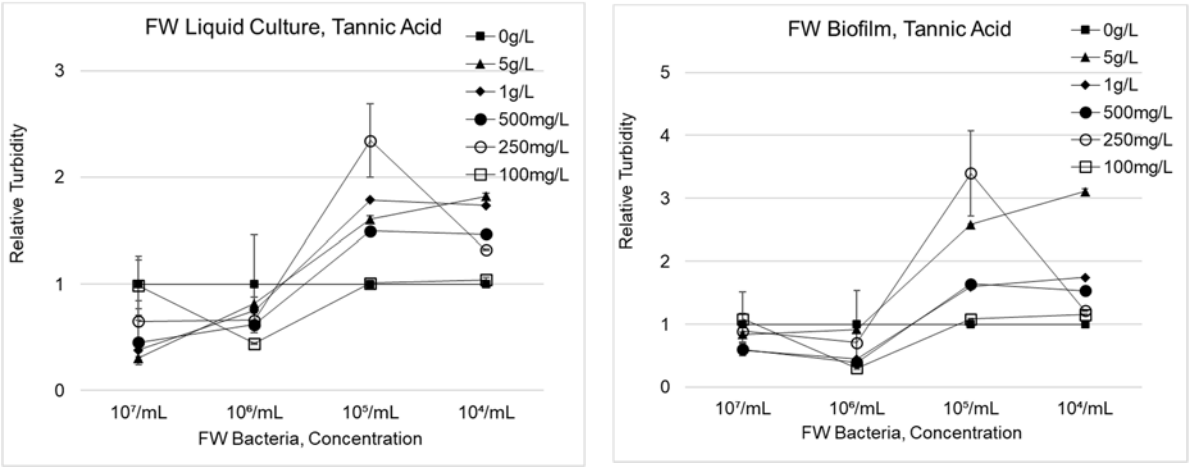

(a) **(b)**

Figure S14. (**a**) The effect of increasing tannic acid concentrations on the growth of FW liquid cultures and (**b**) on the formation of FW biofilms.
